# Supplementary material for: Whole-genome resequencing reveals Brassica napus origin and genetic loci involved in its improvement
Source: Nat Commun. 2019 Mar 11;10:1154. doi: 10.1038/s41467-019-09134-9 (PMC6411957; doi:10.1038/s41467-019-09134-9)
Supplement: Supplementary file 1 — Supplementary Information [file 41467_2019_9134_MOESM1_ESM.pdf]

Whole-genome resequencing reveals *Brassica napus* origin and genetic loci involved in its improvement

Lu *et al.*

Supplementary Figures

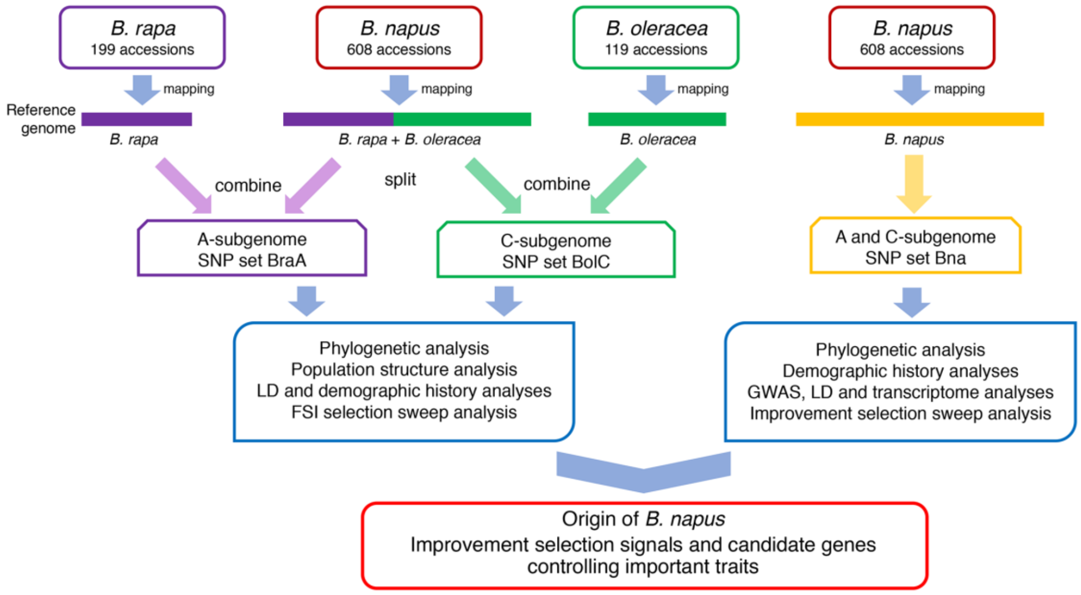

Supplementary Figure 1. Workflow performed in this study.

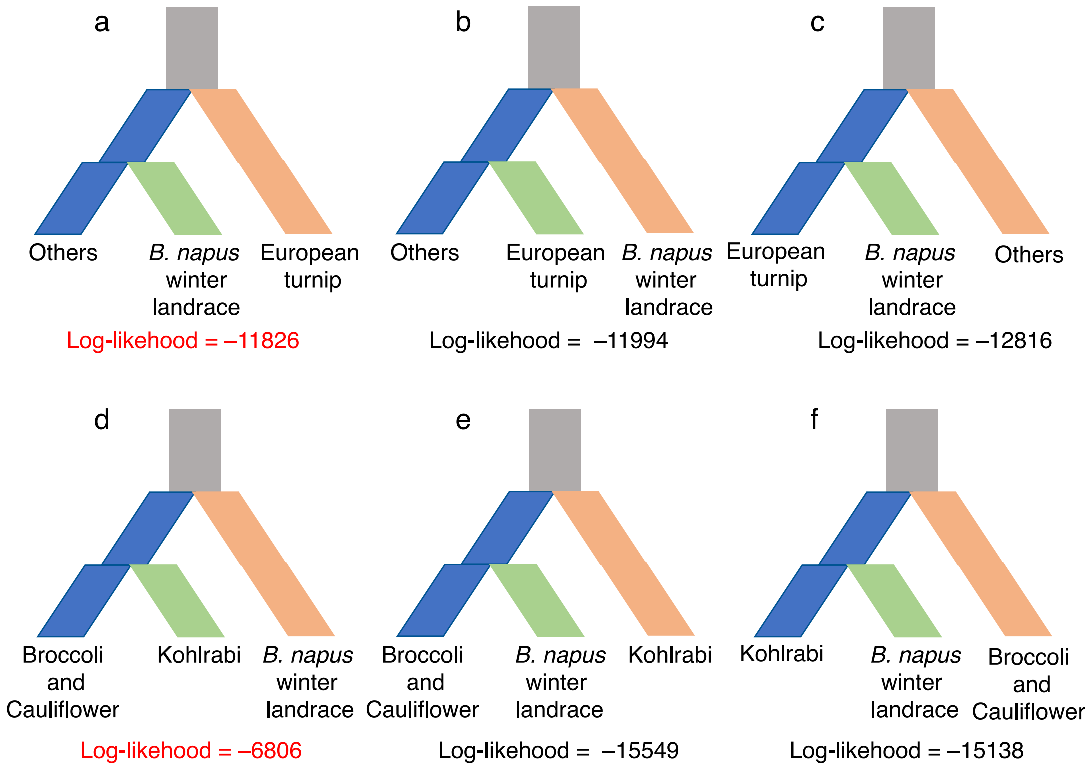

Supplementary Figure 2. The demographic models evaluated with ∂a∂i[1]. The Log-likelihood value is indicated under each model.

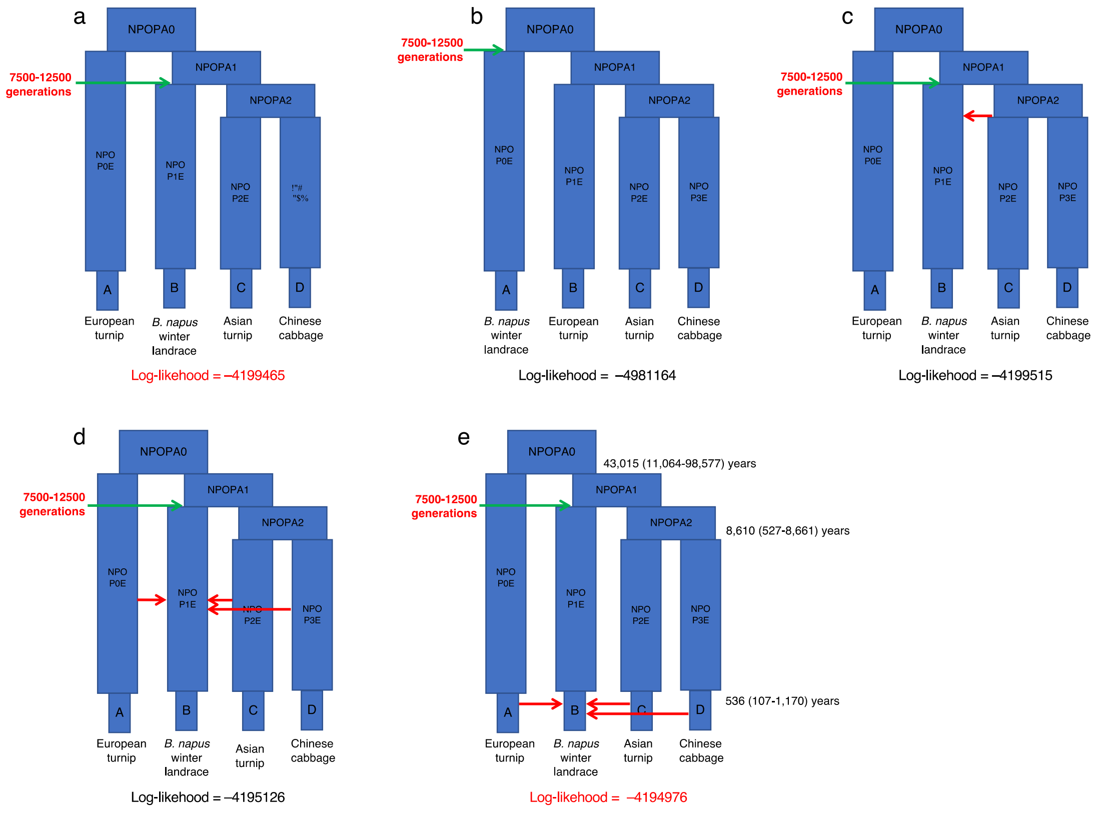

Supplementary Figure 3. The demographic history of the A subgenome of *B. napus*. The Log-likelihood value is indicated under each model. All the red lines indicate the migration events from *B. rapa* into the *B. napus* winter landraces. The generation number of *B. napus* was set to 7500 to 12500[2]. The divergence time for different split events was estimated using fastsimcoal2[3], and marked on the right of the corresponding event. Three deduced ancient populations were marked as NPOPA0 to NPOPA2.

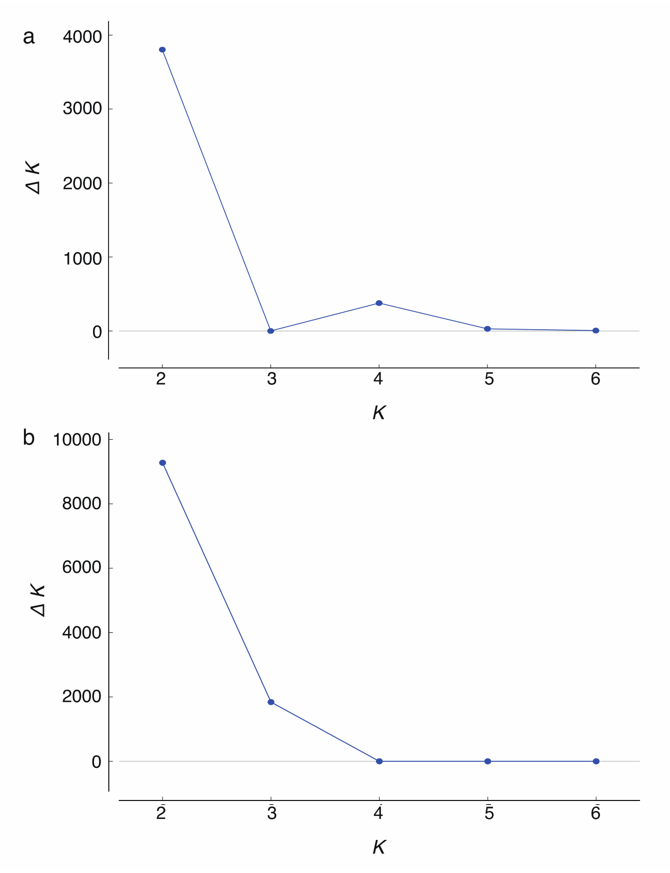

Supplementary Figure 4. *ΔK* based on rate of change of LnP(*K*) between successive K values. (a) Comparison of *ΔK* for population structure of *B. napus* landraces and *B. rapa* accessions; (b) comparison of *ΔK* for population structure of *B. napus* landraces and *B. oleracea* accessions.

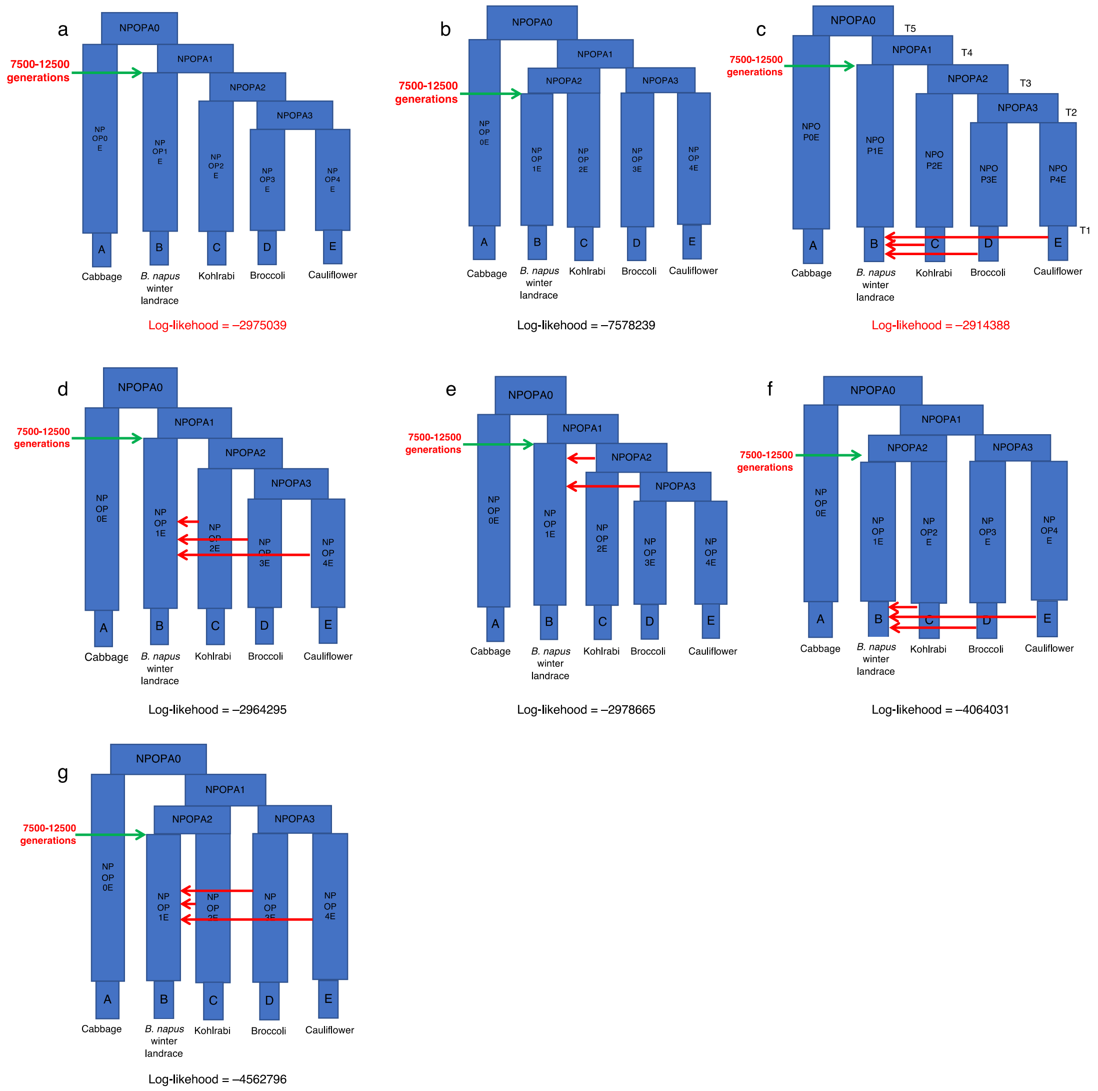

Supplementary Figure 5. Comparison of different demographic models of the C subgenome of *B. napus*. The Log-likelihood value is indicated under each model. No migration was considered in a and b. Recent migration from *B. oleracea* into the *B. napus* landraces was considered in models c and f; middle-term migration from *B. oleracea* into *B. napus* landraces was considered in models d and g; long-term migration from *B. oleracea* into *B. napus* landraces was considered in model e. The generation number of *B. napus* was set to 7500 to 12500[2]. All the red lines indicate the migration events. Four deduced ancient populations were marked as NPOPA0 to NPOPA3.

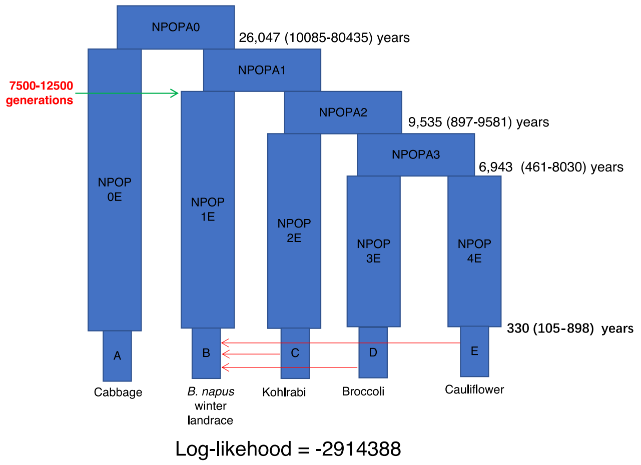

Supplementary Figure 6. The demographic history of the C subgenome of *B. napus*. The Log-likelihood value is indicated under the model. All the red lines indicate the recent migration events from *B. oleracea* into the *B. napus* landraces. The generation number of *B. napus* was set to 7500 to 12500[2]. The divergence time for different split events was estimated using fastsimcoal2[3] and marked on the right of the corresponding event. Four deduced ancient populations were marked as NPOPA0 to NPOPA3.

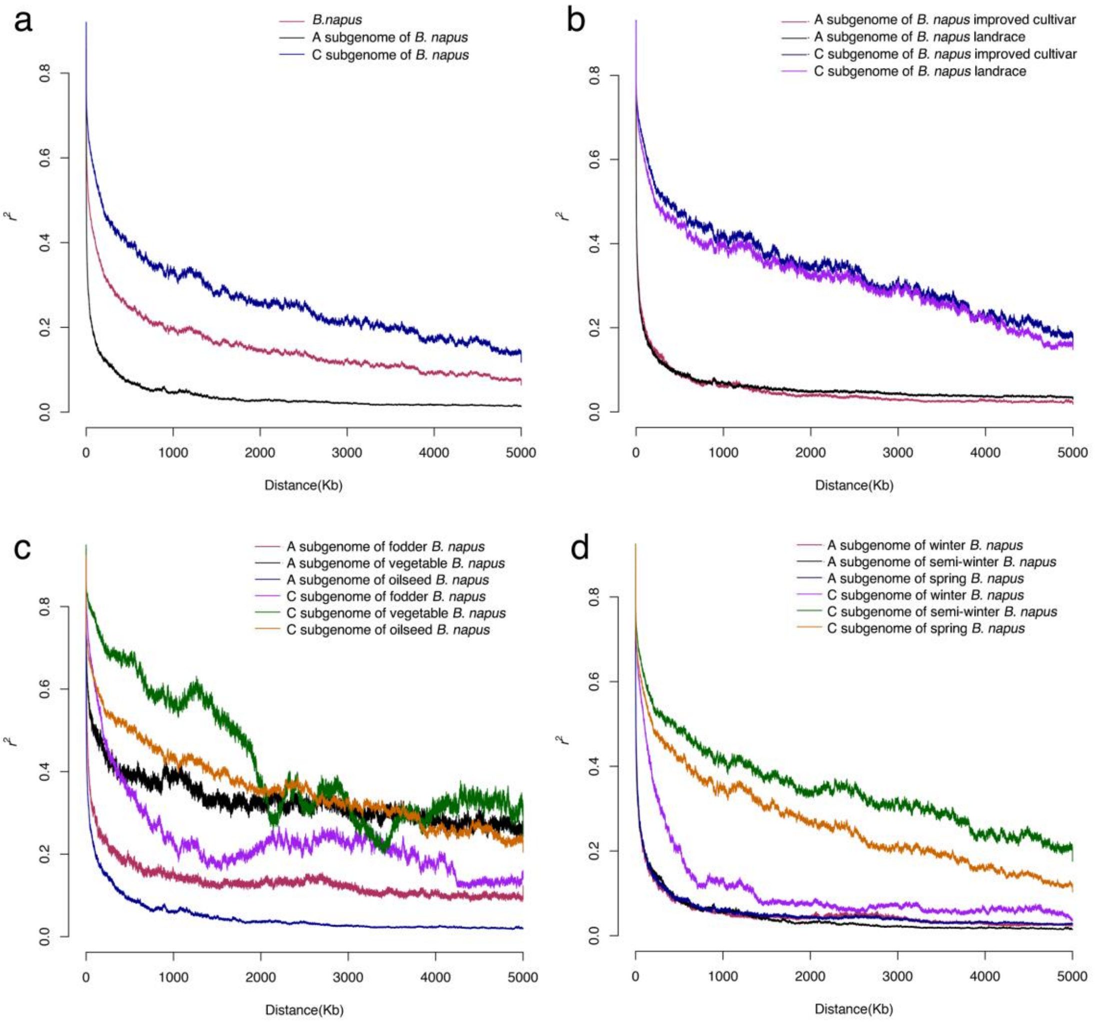

Supplementary Figure 7. Comparison of LD decay at the genome- and subgenome-wide level. (a) Comparison of genome- and subgenome-wide LD decay for the *B. napus* accessions. The LD decay from the A and C subgenomes is indicated by black and red lines, respectively, and that from the *B. napus* genome is indicated in blue. (b) Comparison of subgenome-wide LD decay for *B. napus* landraces and improved cultivars. The LD decay from the A and C subgenomes of *B. napus* landraces is indicated by black and blue-violet lines, and that from *B. napus* improved cultivars is indicated by red and navy blue lines, respectively. (c) Comparison of subgenome-wide LD decay for *B. napus* with different uses. The LD decay from the

A subgenome of *B. napus* accessions cultivated for fodder, vegetable, and oil purposes is indicated by red, black, and navy blue lines, and that from the C subgenome is indicated by blue-violet, dark green, and brown lines, respectively. (d) Comparison of subgenome-wide LD decay for *B. napus* with different ecotypes. The LD decay from the A subgenome of *B. napus* for winter, semi-winter, and spring ecotypes is indicated by red, black, and navy blue lines, and that from the C subgenome by blue-violet, dark green, and brown lines, respectively. The LD decay of different groups of accessions was calculated using the Bna SNP set.

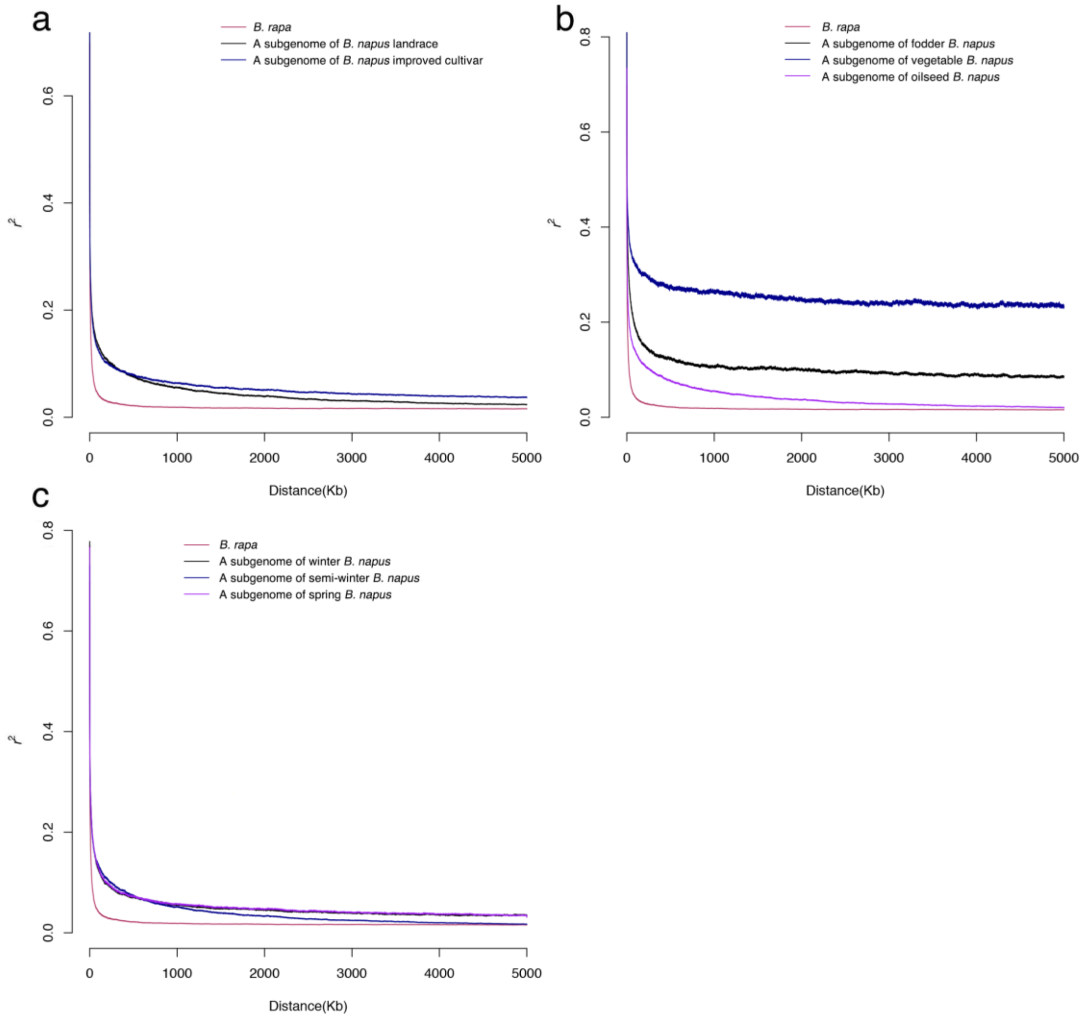

Supplementary Figure 8. Comparison of LD decay based on BraA SNP sets. (a) LD decay of *B. rapa*, and the A subgenomes of *B. napus* landraces and improved cultivars. (b) LD decay of *B. rapa*, and the A subgenomes of *B. napus* with oil, vegetable, and fodder purposes, respectively. (c) LD decay of *B. rapa*, and the A subgenomes of *B. napus* with winter, semi-winter, and spring ecotypes, respectively. The LD decay of different groups of accessions was calculated using the BraA SNP set.

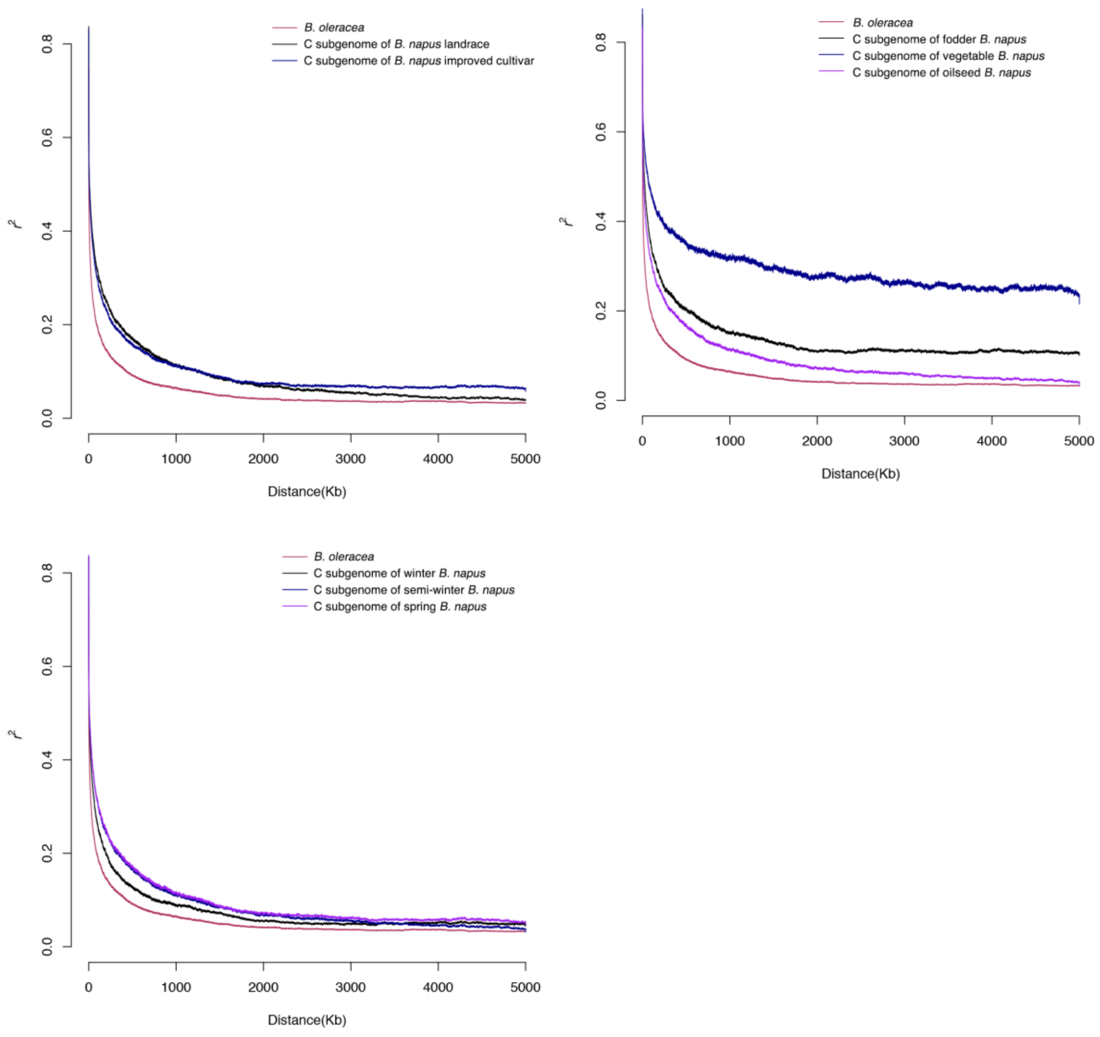

Supplementary Figure 9. Comparison of LD decay based on BolC SNP sets. (a) LD decay of *B. oleracea*, and the C subgenomes of *B. napus* landraces and improved cultivars. (b) LD decay of *B. oleracea*, and the C subgenomes of *B. napus* with oil, vegetable, and fodder purposes, respectively. (c) LD decay of *B. oleracea*, and the C subgenomes of *B. napus* with winter, semi-winter, and spring ecotypes, respectively. The LD decay of different groups of accessions was calculated using the BolC SNP set.

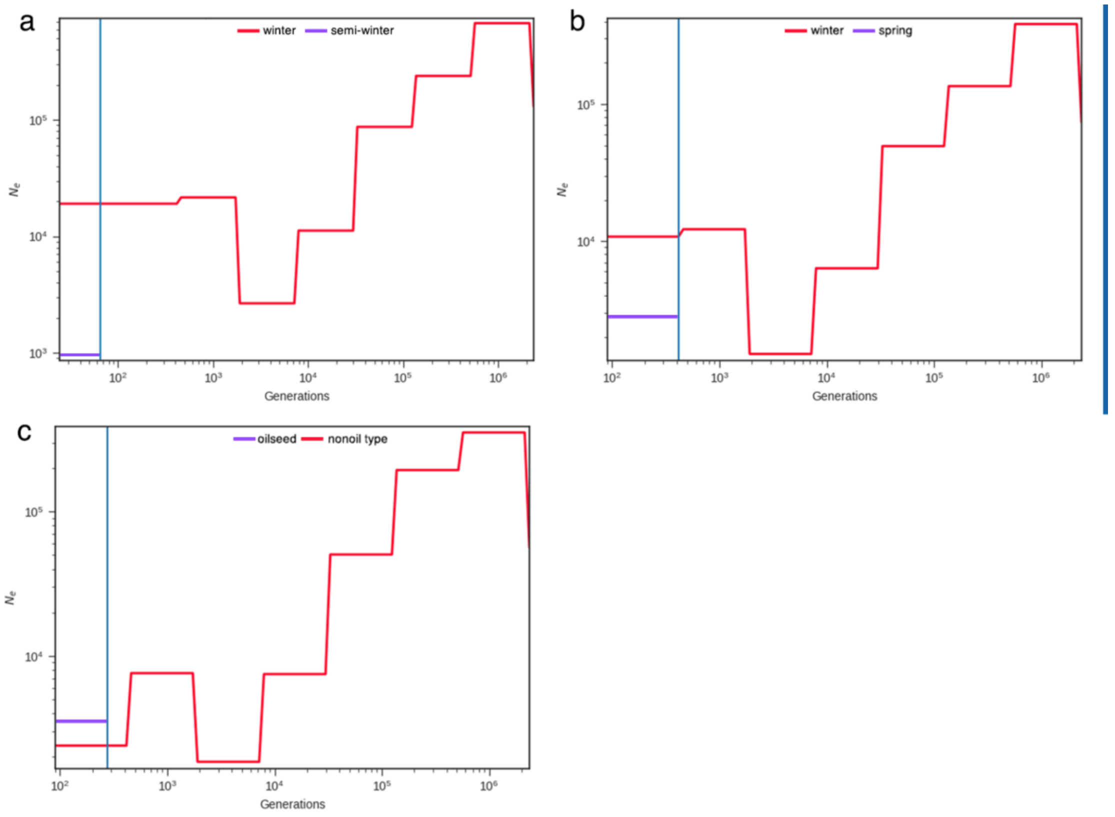

Supplementary Figure 10. Divergence history of different ecotype and usage of *B. napus*. (a) Divergence between *B. napus* winter and semi-winter ecotype; (b) Divergence between *B. napus* winter and spring ecotype; (c) Divergence between oilseed and non-oilseed (Combined fodder and vegetable accessions) *B. napus*. The historical effective population sizes (*Ne*) and divergence time were estimated using SMC++[4]. Generation estimates were inferred by assuming that the upper and lower mutation rates were 1.5×10^{-8} and 9×10^{-9} per synonymous site per generation, respectively, and that the generation time was one year.

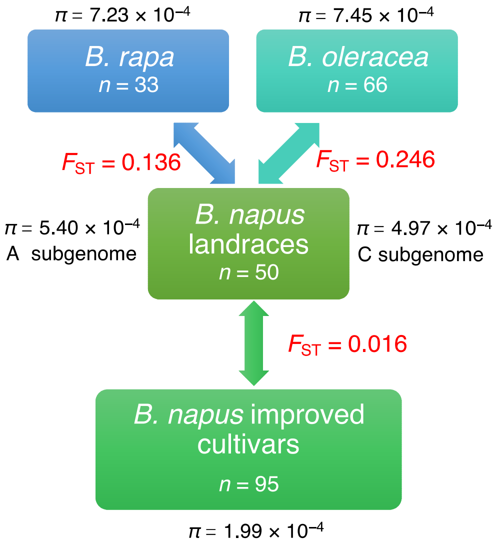

Supplementary Figure 11. Genome- and subgenome-wide comparison of nucleotide diversity and F_{ST} during the FSI and SSI of *B. napus*.

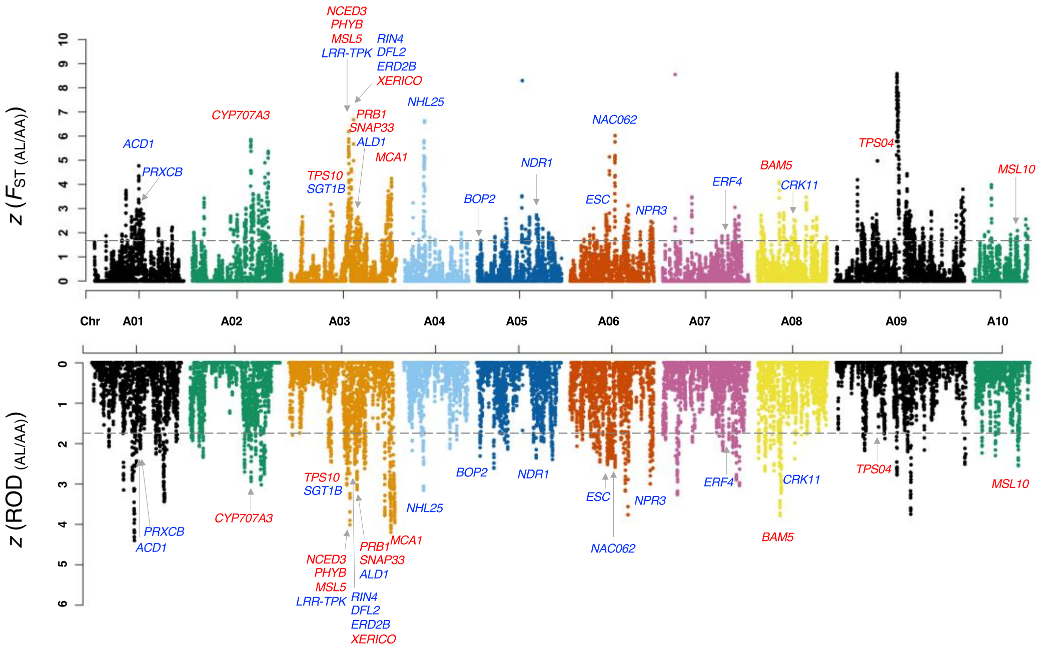

Supplementary Figure 12. Whole-genome analysis of the selection signatures in A subgenome during the FSI of *B. napus*. The upper and lower panels show the genome-wide screening of FSI-selection signals of $F_{ST\ (AL/AA)}$ and ROD $_{(AL/AA)}$, respectively. Horizontal dashed lines show the significance level of α = 0.05, corresponding to z = 1.645. Genes involved in abiotic response are labeled in red; disease defense response genes are labeled in blue. Full descriptions of these genes are shown in Supplementary Data 29.

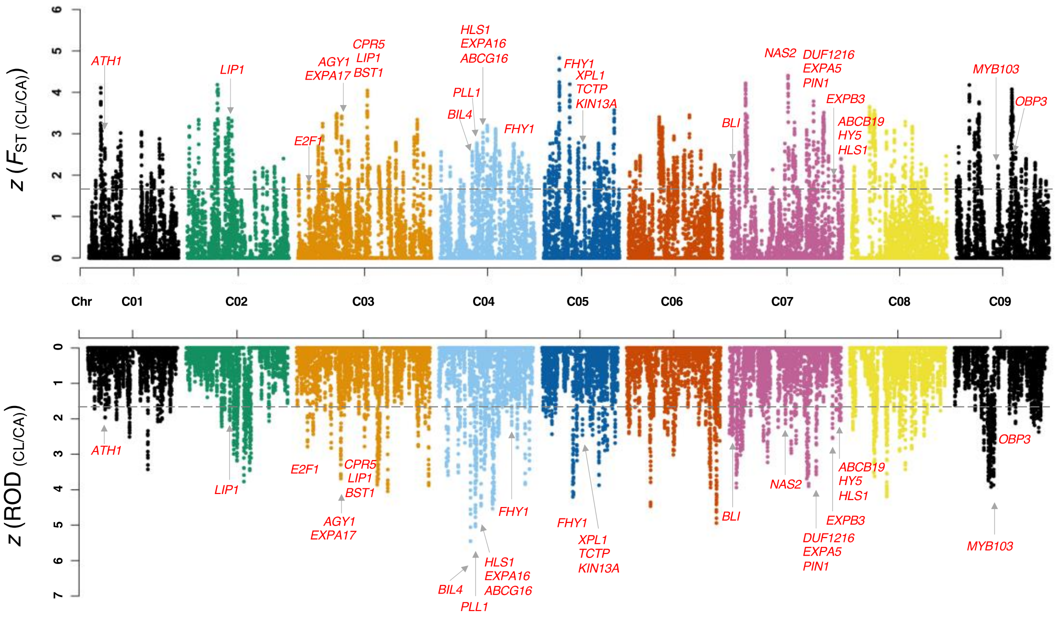

Supplementary Figure 13. Whole-genome analysis of the selection signatures in C subgenome during the FSI of *B. napus*. The upper and lower panels show the genome-wide screening of FSI-selection signals of F_{ST} (CL/CA) and ROD (CL/CA), respectively. Horizontal dashed lines show the significance level of α = 0.05, corresponding to z = 1.645. Genes involved in morphogenesis are labeled in red. Full descriptions of these genes are shown in Supplementary Data 29.

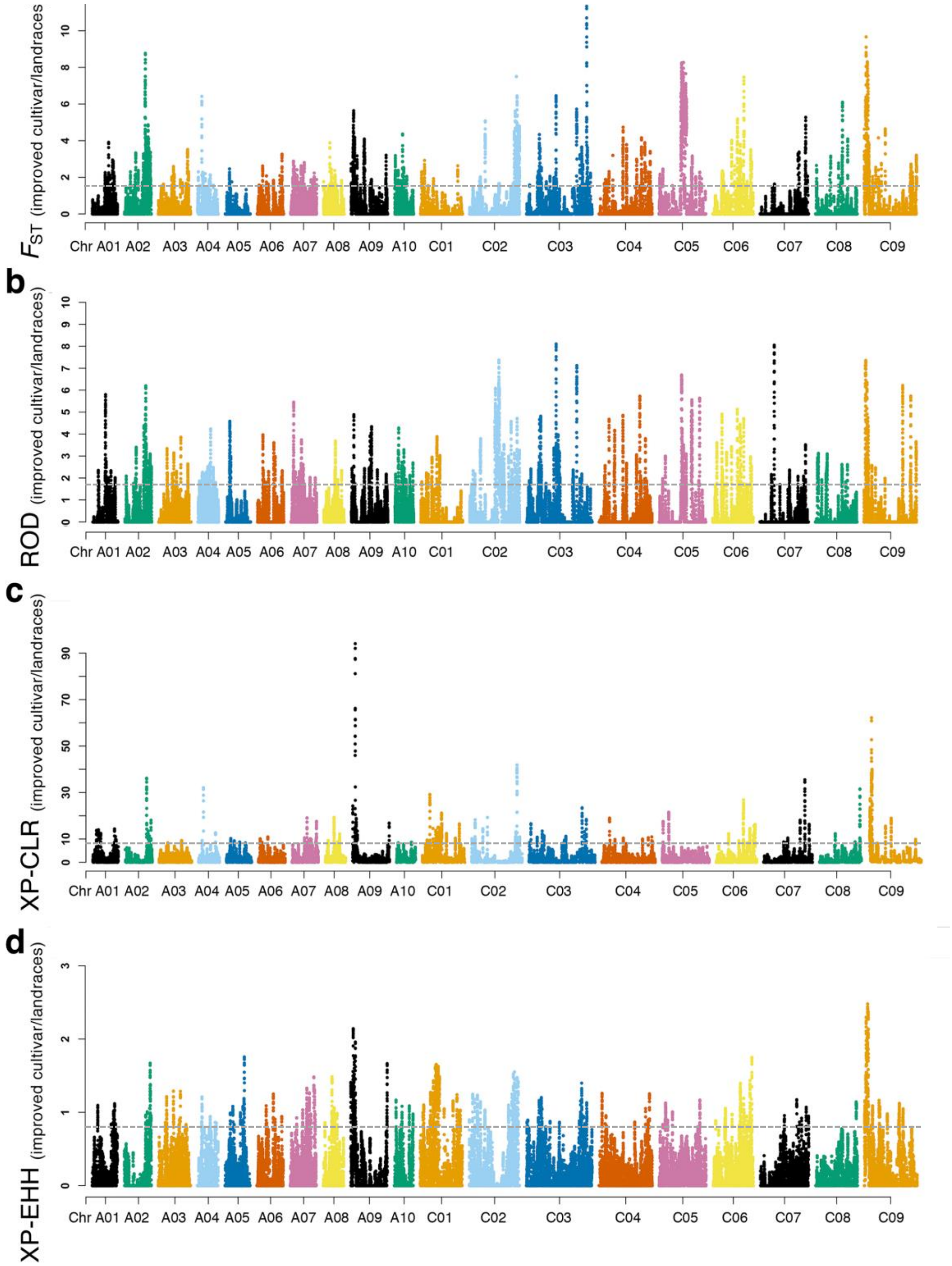

Supplementary Figure 14. Whole-genome scanning of the improvement-selection signatures between *B. napus* improved cultivars and landraces. Improvement-selection signals detected using (a) F_{ST}, (b) ROD, (c) XPCLR, and (d) XP-EHH comparisons.

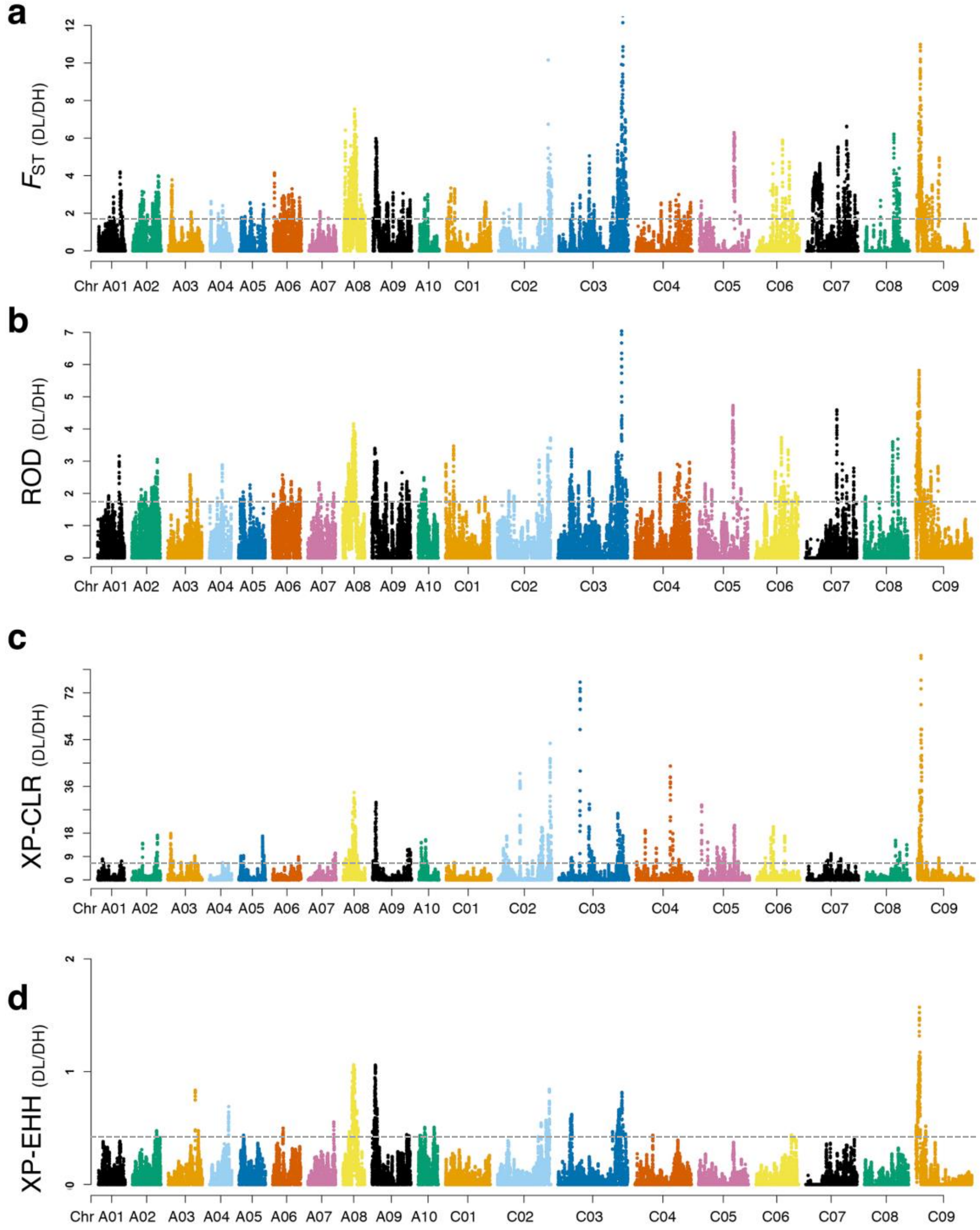

Supplementary Figure 15. Whole-genome scanning of the improvement-selection signatures between double-low (DL) and double high (DH) *B. napus* cultivars. Improvement-selection signals detected using (a) F_{ST}, (b) ROD, (c) XPCLR, and (d) XP-EHH comparisons.

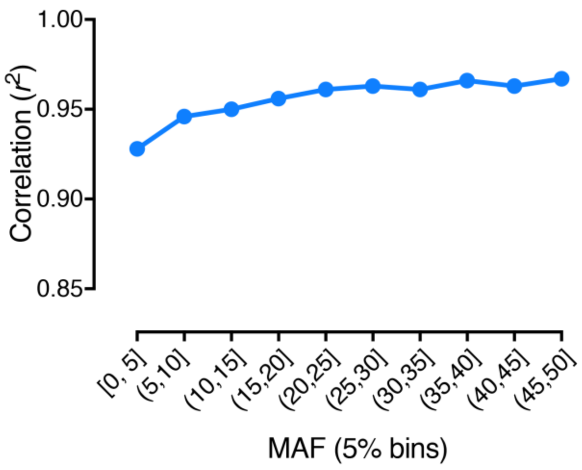

Supplementary Figure 16. Evaluation of imputation accuracy. Correlations (r^2) between true and imputed genotypes were calculated at each locus for two biological replicates of 20 *B. napus* accessions in intervals of 5% of the MAF. Missing SNPs in the true genotypes were excluded when calculating the correlations.

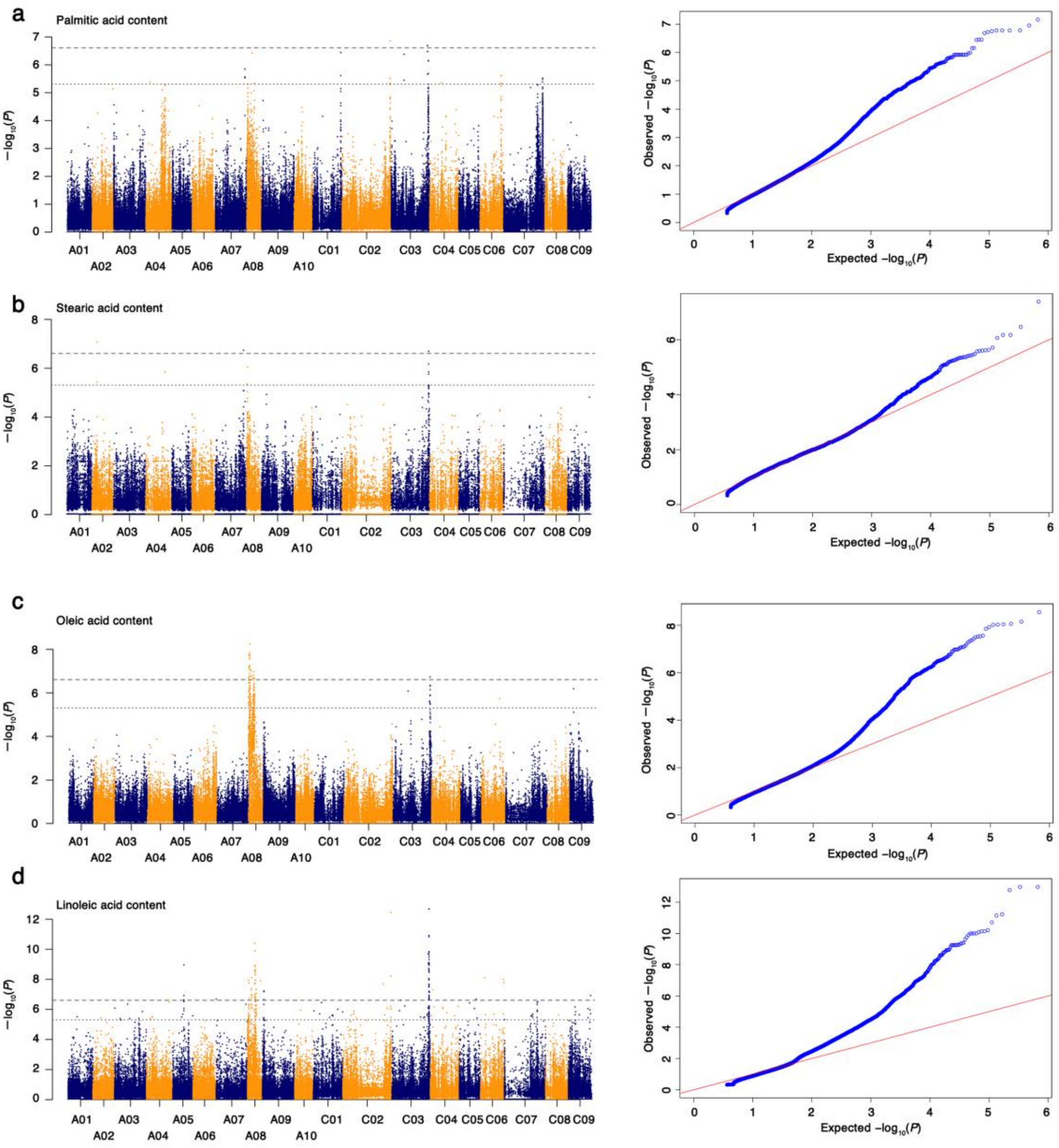

continued on next page

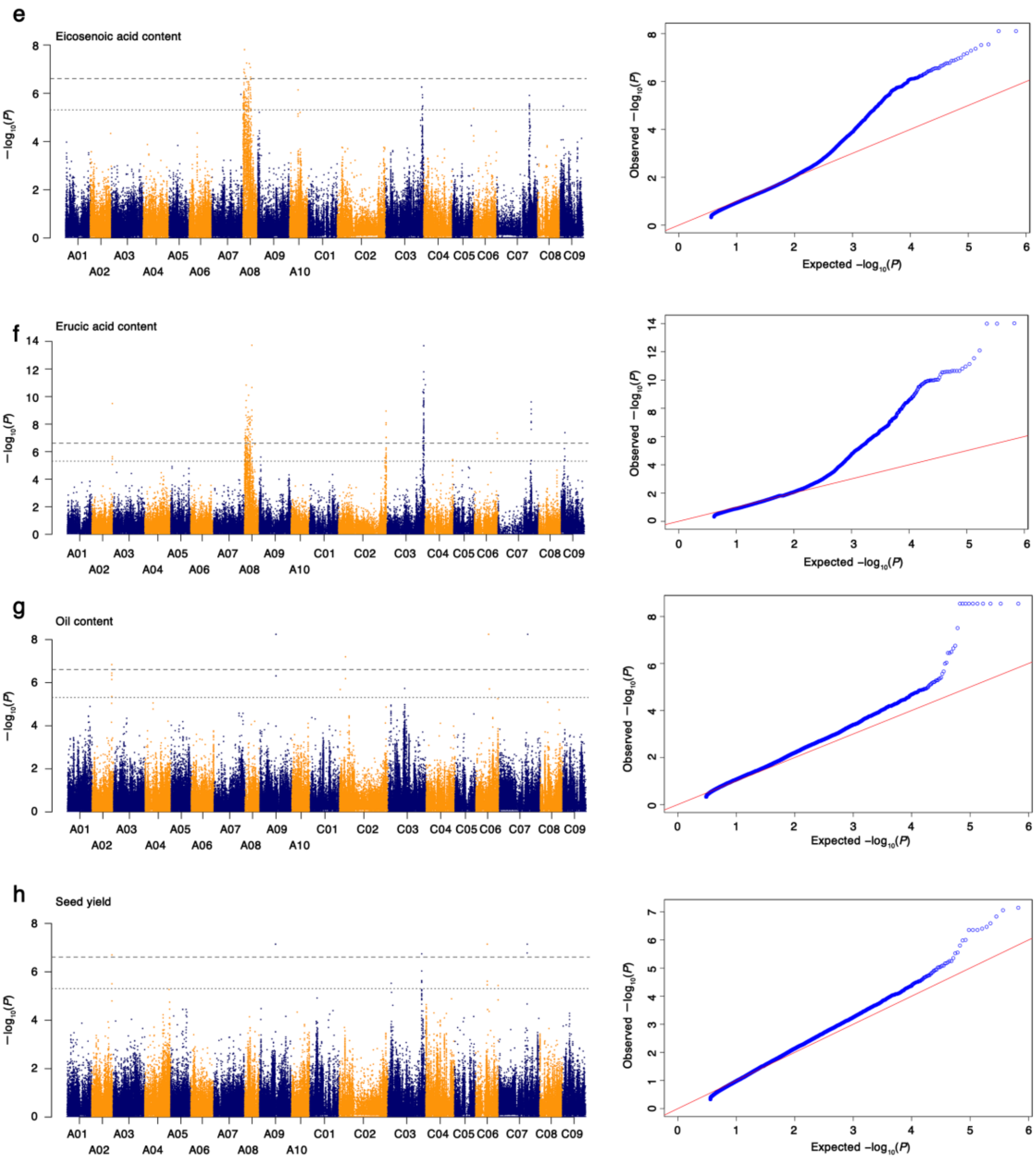

continued on next page

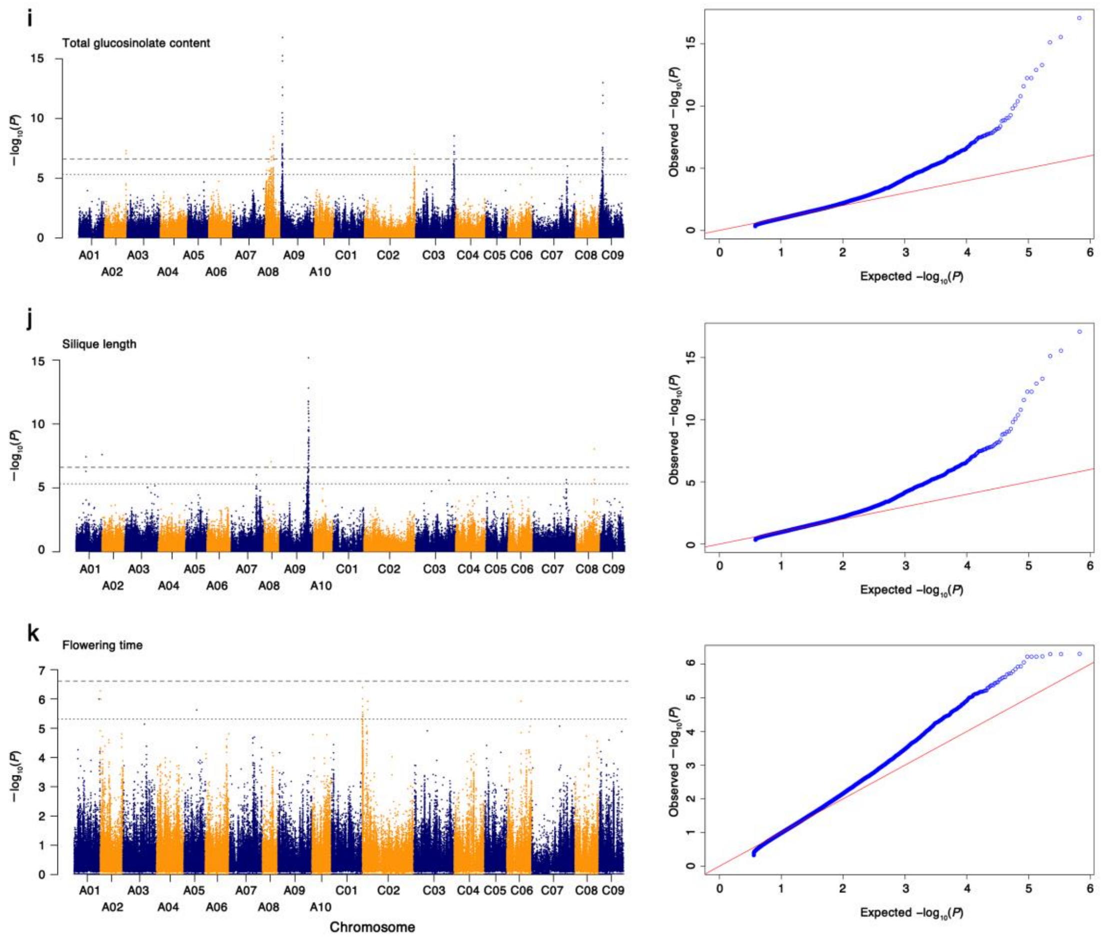

Supplementary Figure 17. Manhattan and quantile-quantile (QQ) plots of GWAS for BLUP of 11 traits. (a) Palmitic acid content; (b) stearic acid content; (c) oleic acid content; (d) linoleic acid content; (e) eicosenoic acid content; (f) erucic acid content; (g) oil content; (h) seed yield; (i) total glucosinolate content; (j) silique length; and (k) flowering time. The lower and upper dashed horizontal lines depict the suggestive (−log10(*P*) = 5.31) and significance thresholds (−log10(*P*) = 6.61), respectively.

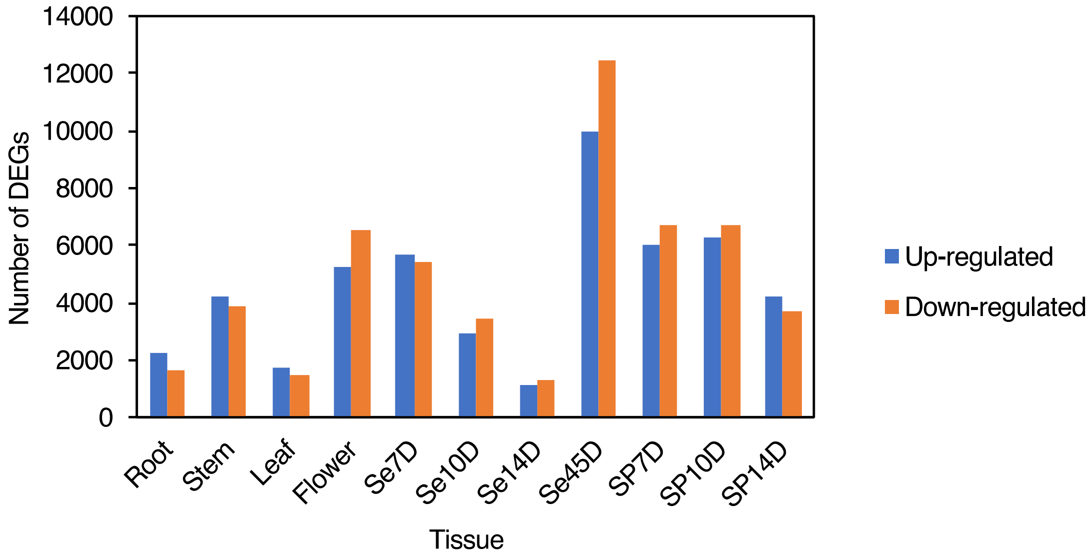

Supplementary Figure 18. Number of DEGs in the indicated tissues between double-high and double-low *B. napus* accessions.

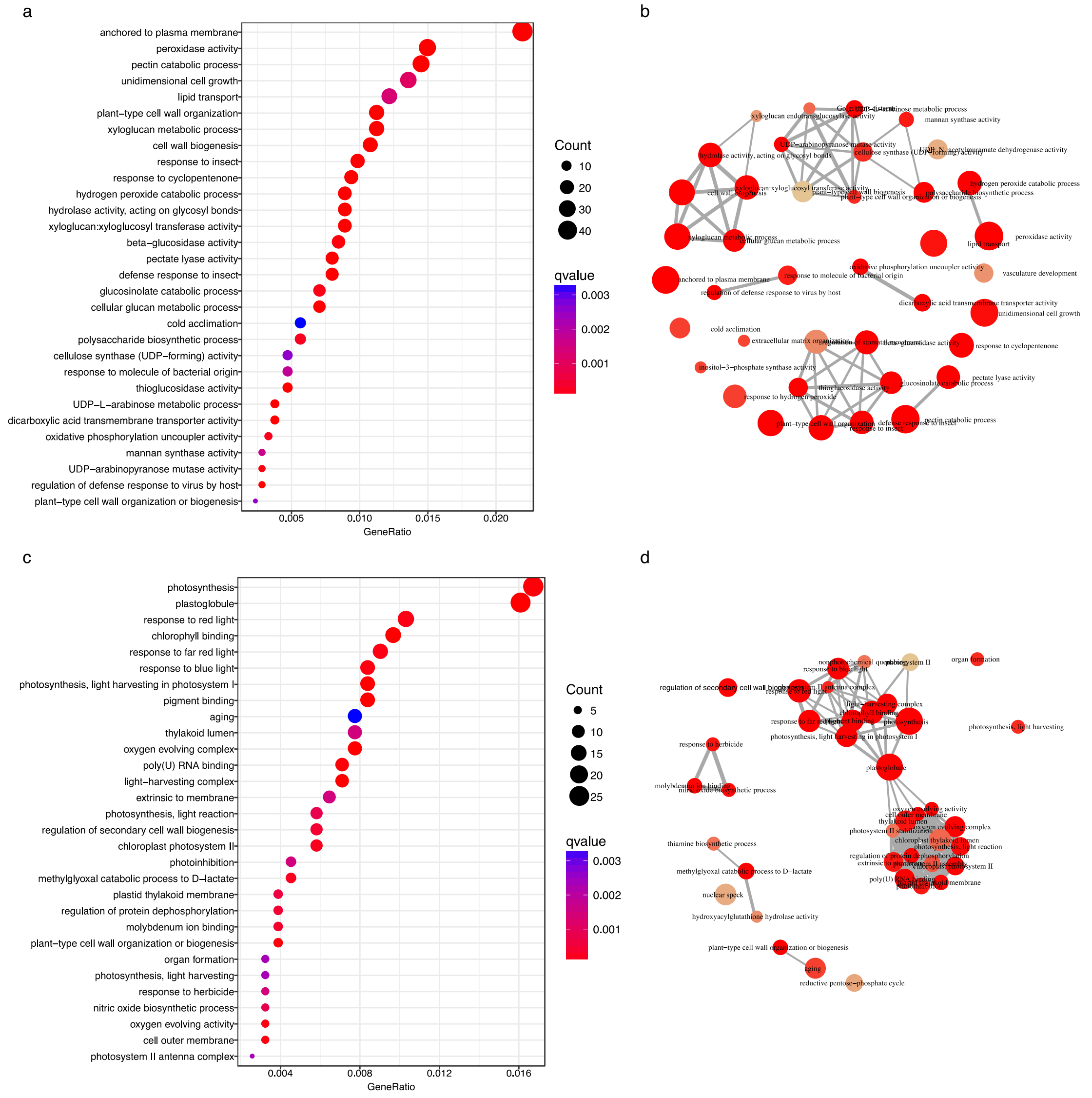

Supplementary Figure 19. GO enrichment analysis results of DEGs between roots of double-high and double-low *B. napus* accessions. (a) and (c) Overrepresented GO terms in up- and down-regulated DEGs, respectively. (b) and (d) Enrichment results of up- and down-regulated DEGs, respectively. Only the top 30 significantly enriched GO terms were included in this figure.

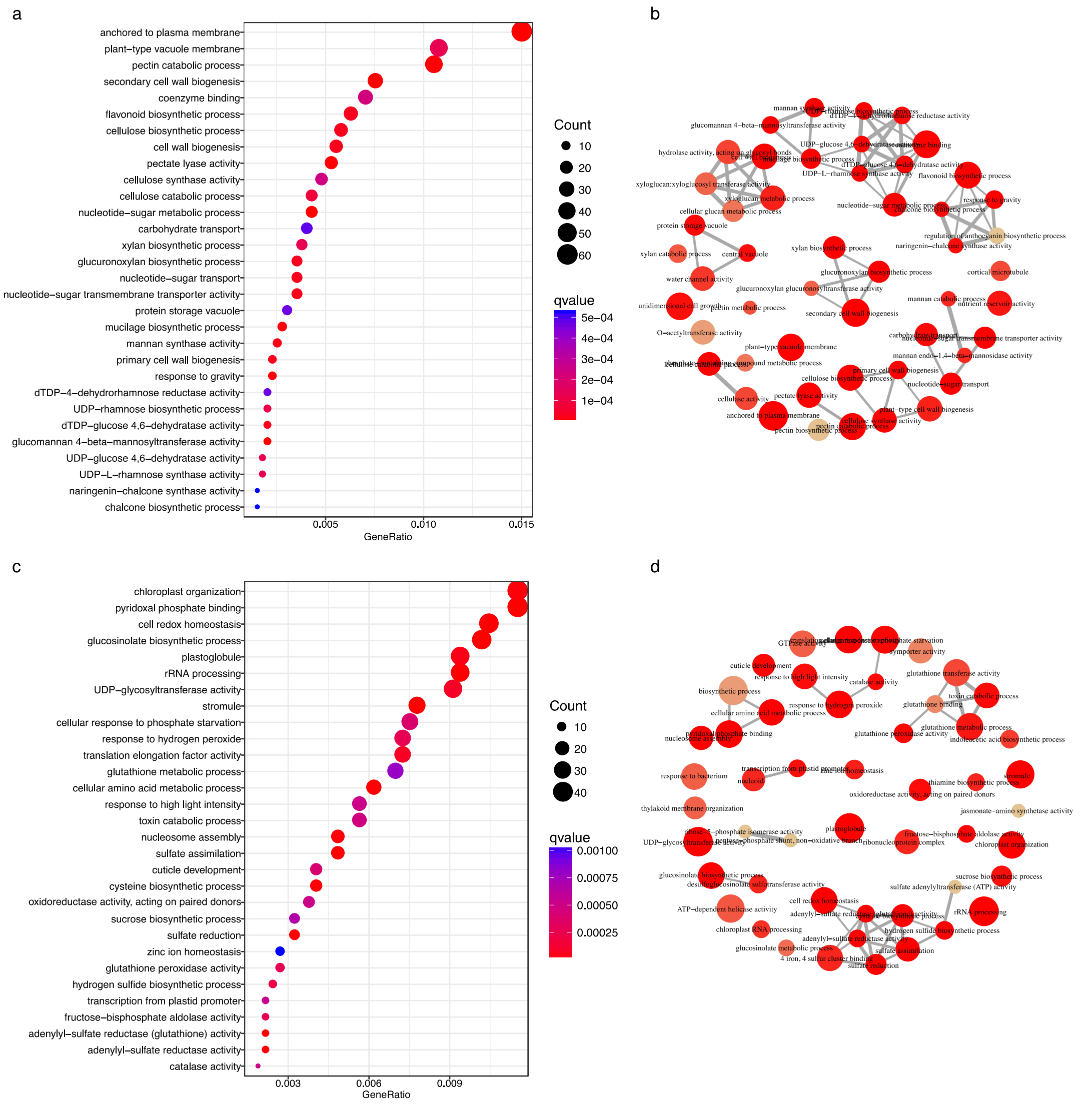

Supplementary Figure 20. GO enrichment analysis results of DEGs between stems of double-high and double-low *B. napus* accessions. (a) and (c) Overrepresented GO terms in up- and down-regulated DEGs, respectively. (b) and (d) Enrichment results of up- and down-regulated DEGs, respectively. Only the top 30 significantly enriched GO terms were included in this figure.

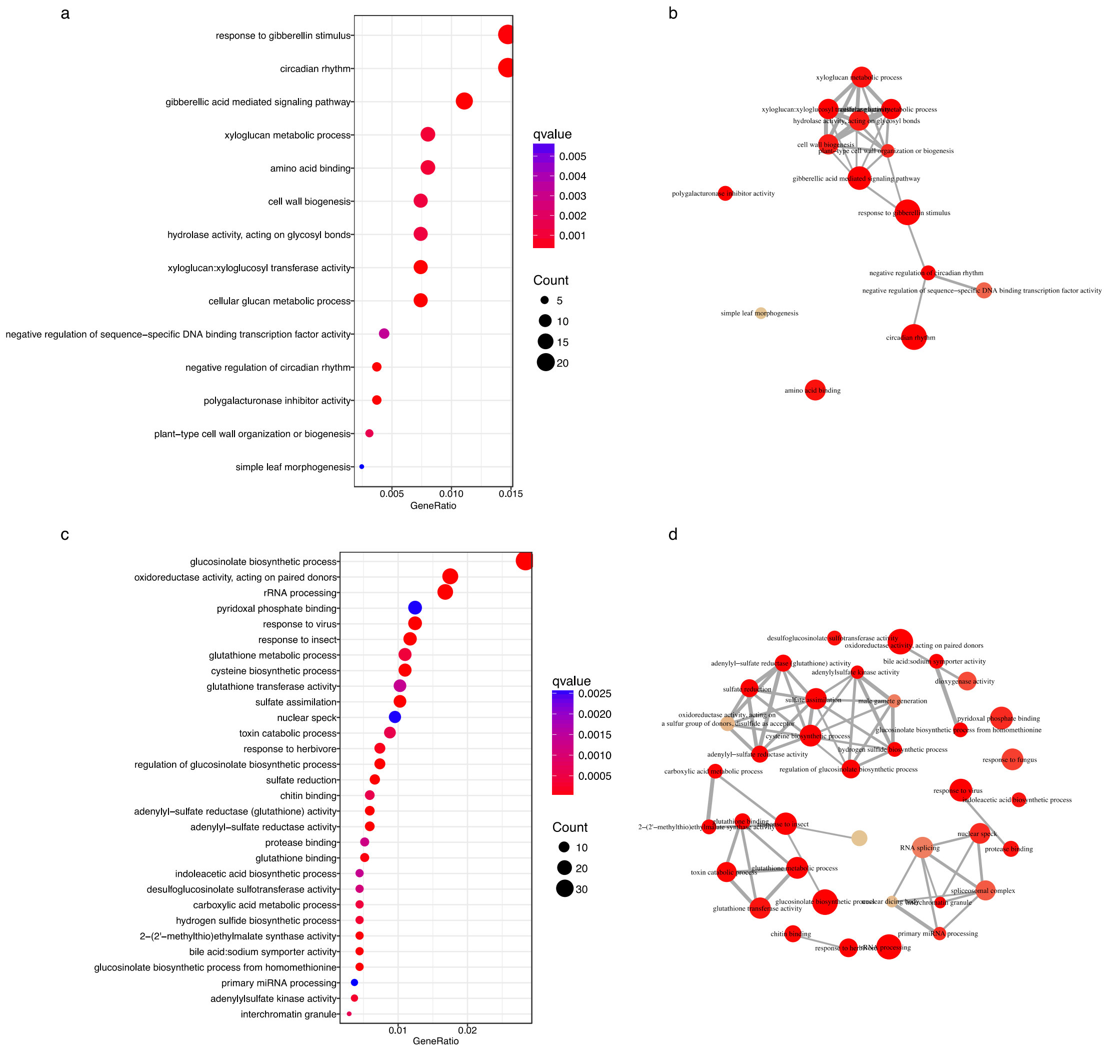

Supplementary Figure 21. GO enrichment analysis results of DEGs between leaves of double-high and double-low *B. napus* accessions. (a) and (c) Overrepresented GO terms in up- and down-regulated DEGs, respectively. (b) and (d) Enrichment results of up- and down-regulated DEGs, respectively. Only the top 30 significantly enriched GO terms were included in this figure.

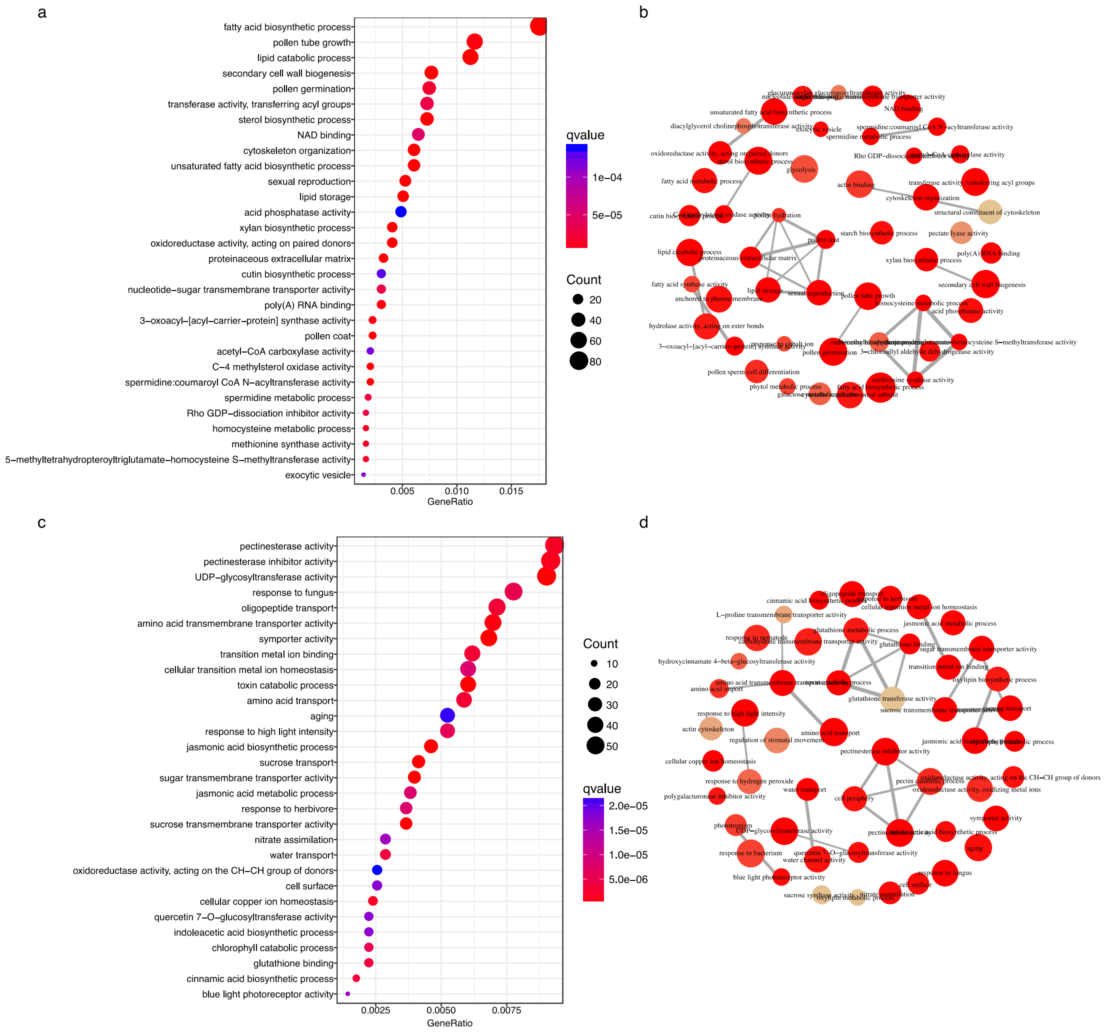

Supplementary Figure 22. GO enrichment analysis results of DEGs between flowers of double-high and double-low *B. napus* accessions. (a) and (c) Overrepresented GO terms in up- and down-regulated DEGs, respectively. (b) and (d) Enrichment results of up- and down-regulated DEGs, respectively. Only the top 30 significantly enriched GO terms were included in this figure.

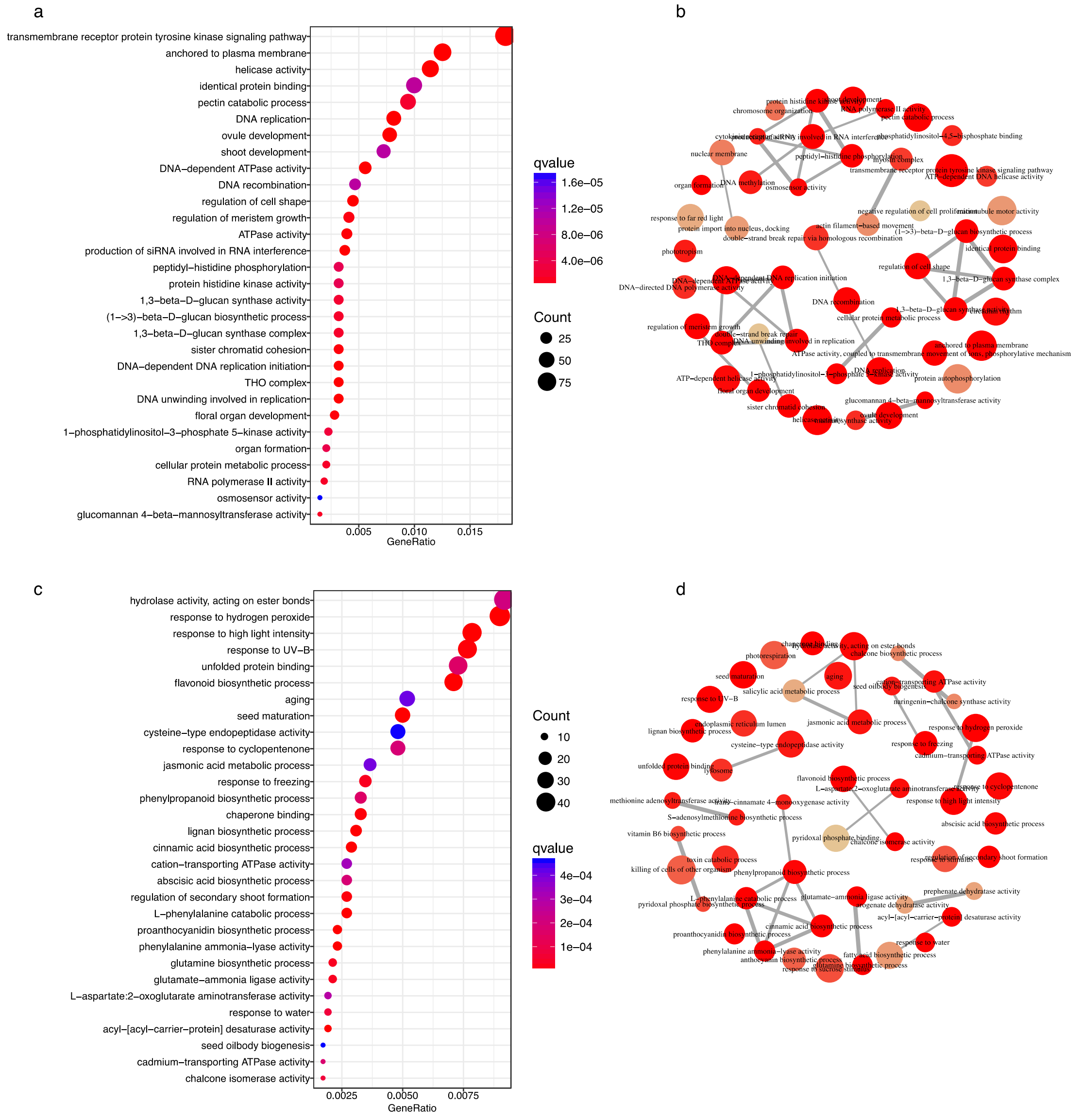

Supplementary Figure 23. GO enrichment analysis results of DEGs between seeds at 7 days after flowering of double-high and double-low *B. napus* accessions. (a) and (c) Overrepresented GO terms in up- and down-regulated DEGs, respectively. (b) and (d) Enrichment results of up- and down-regulated DEGs, respectively. Only the top 30 significantly enriched GO terms were included in this figure.

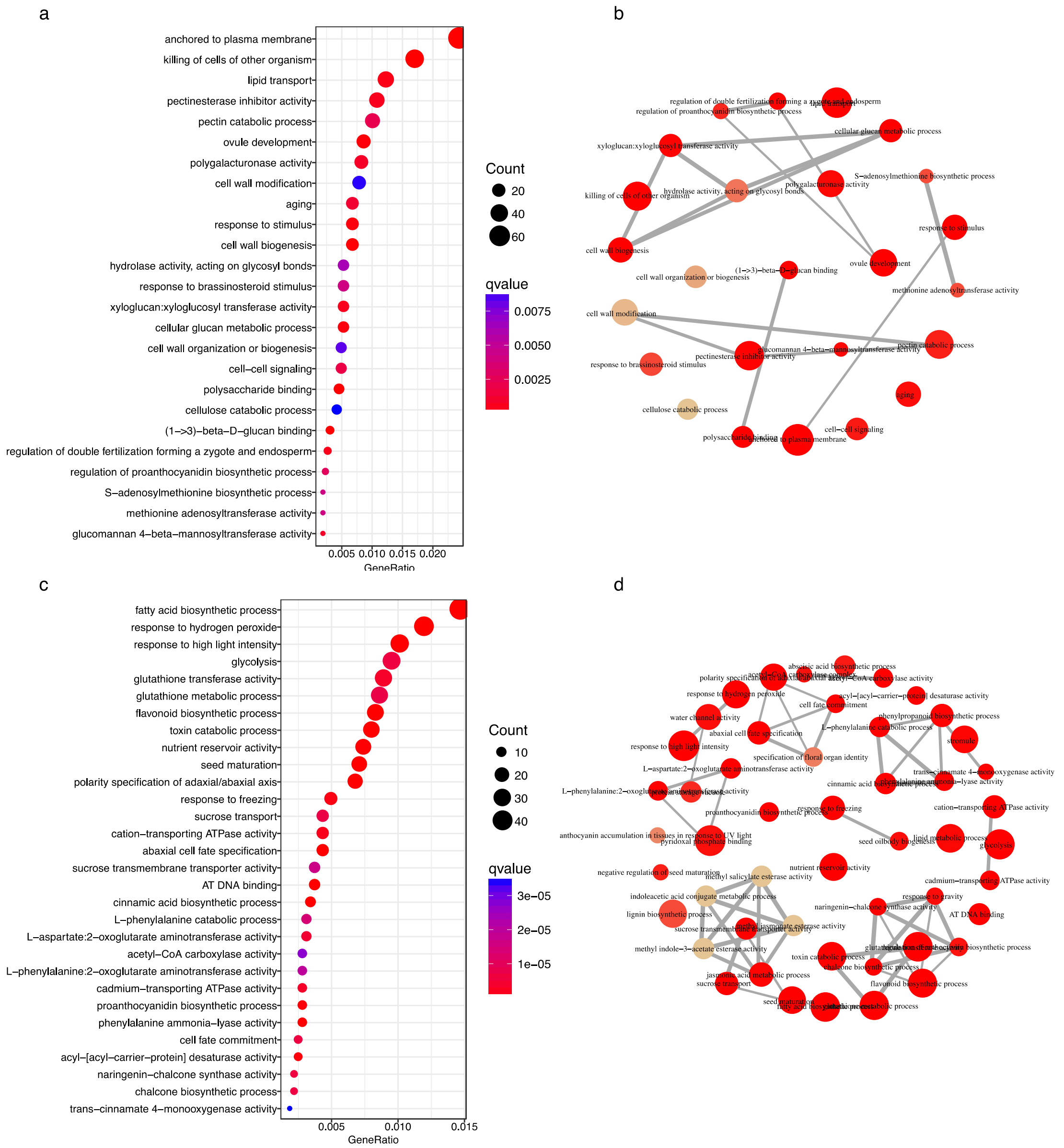

Supplementary Figure 24. GO enrichment analysis results of DEGs between seeds at 10 days after flowering of double-high and double-low *B. napus* accessions. (a) and (c) Overrepresented GO terms in up- and down-regulated DEGs, respectively. (b) and (d) Enrichment results of up- and down-regulated DEGs, respectively. Only the top 30 significantly enriched GO terms were included in this figure.

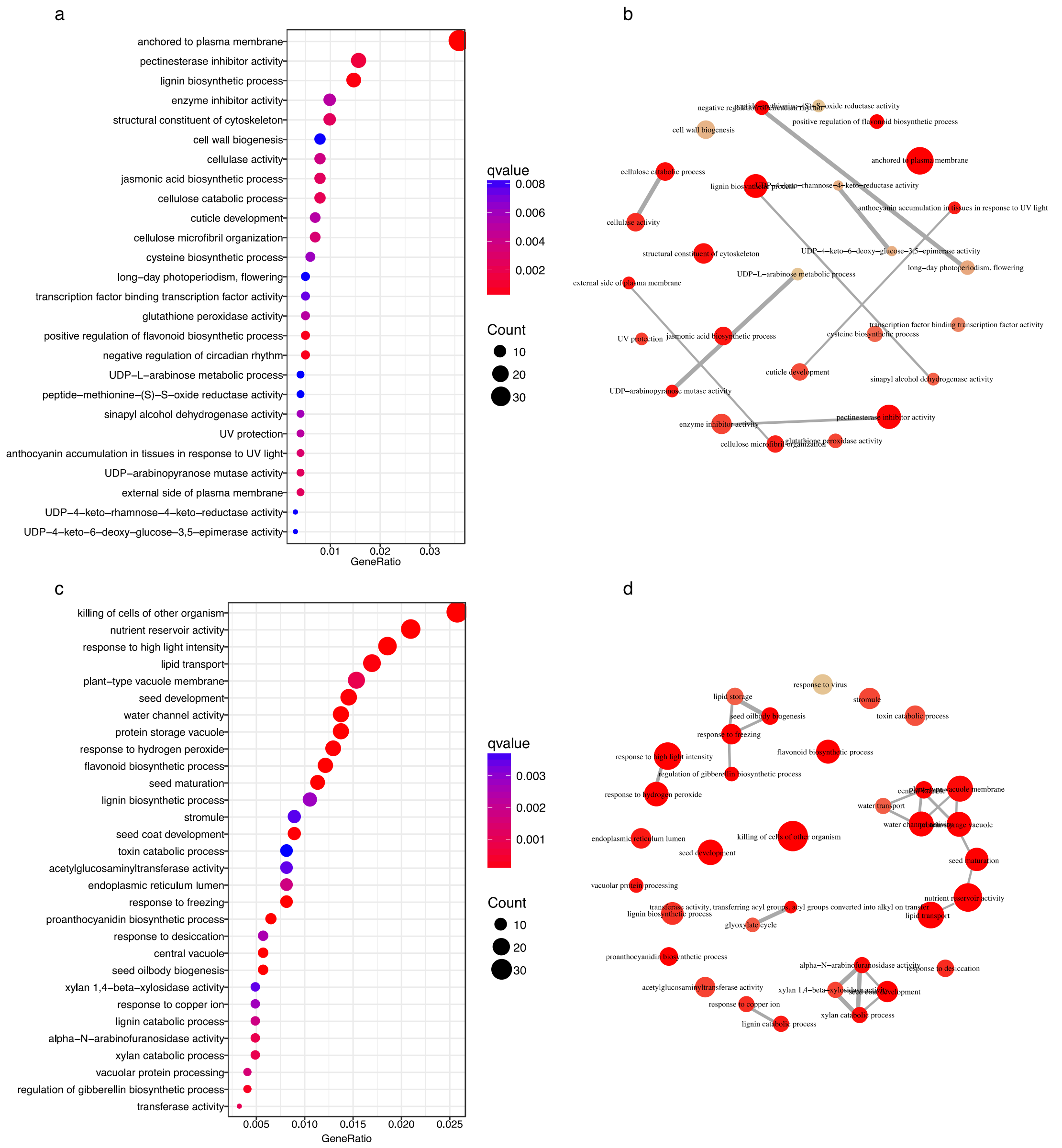

Supplementary Figure 25. GO enrichment analysis results of DEGs between seeds at 14 days after flowering of double-high and double-low *B. napus* accessions. (a) and (c) Overrepresented GO terms in up- and down-regulated DEGs, respectively. (b) and (d) Enrichment results of up- and down-regulated DEGs, respectively. Only the top 30 significantly enriched GO terms were included in this figure.

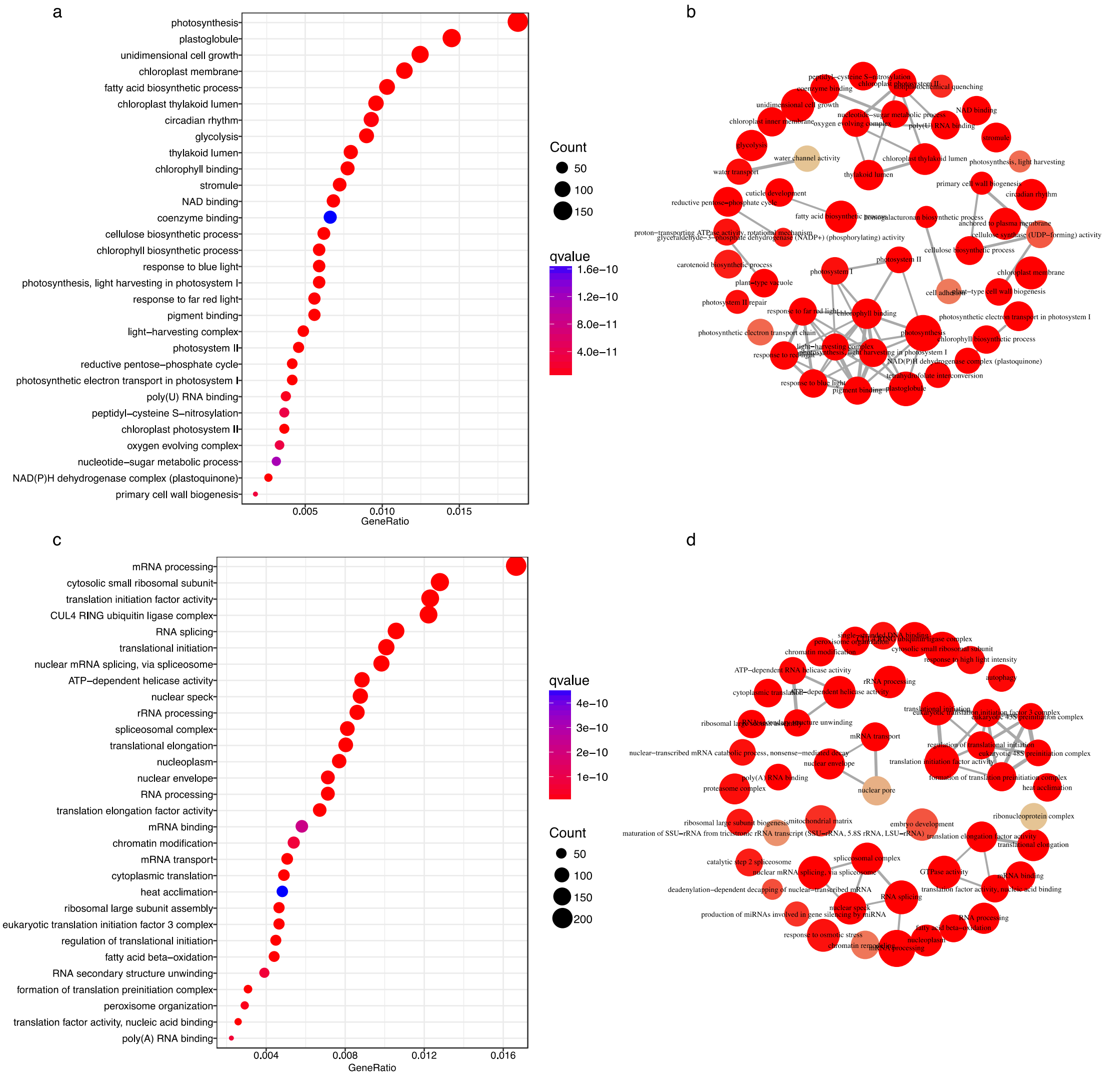

Supplementary Figure 26. GO enrichment analysis results of DEGs between seeds at 45 days after flowering of double-high and double-low *B. napus* accessions. (a) and (c) Overrepresented GO terms in up- and down-regulated DEGs, respectively. (b) and (d) Enrichment results of up- and down-regulated DEGs, respectively. Only the top 30 significantly enriched GO terms were included in this figure.

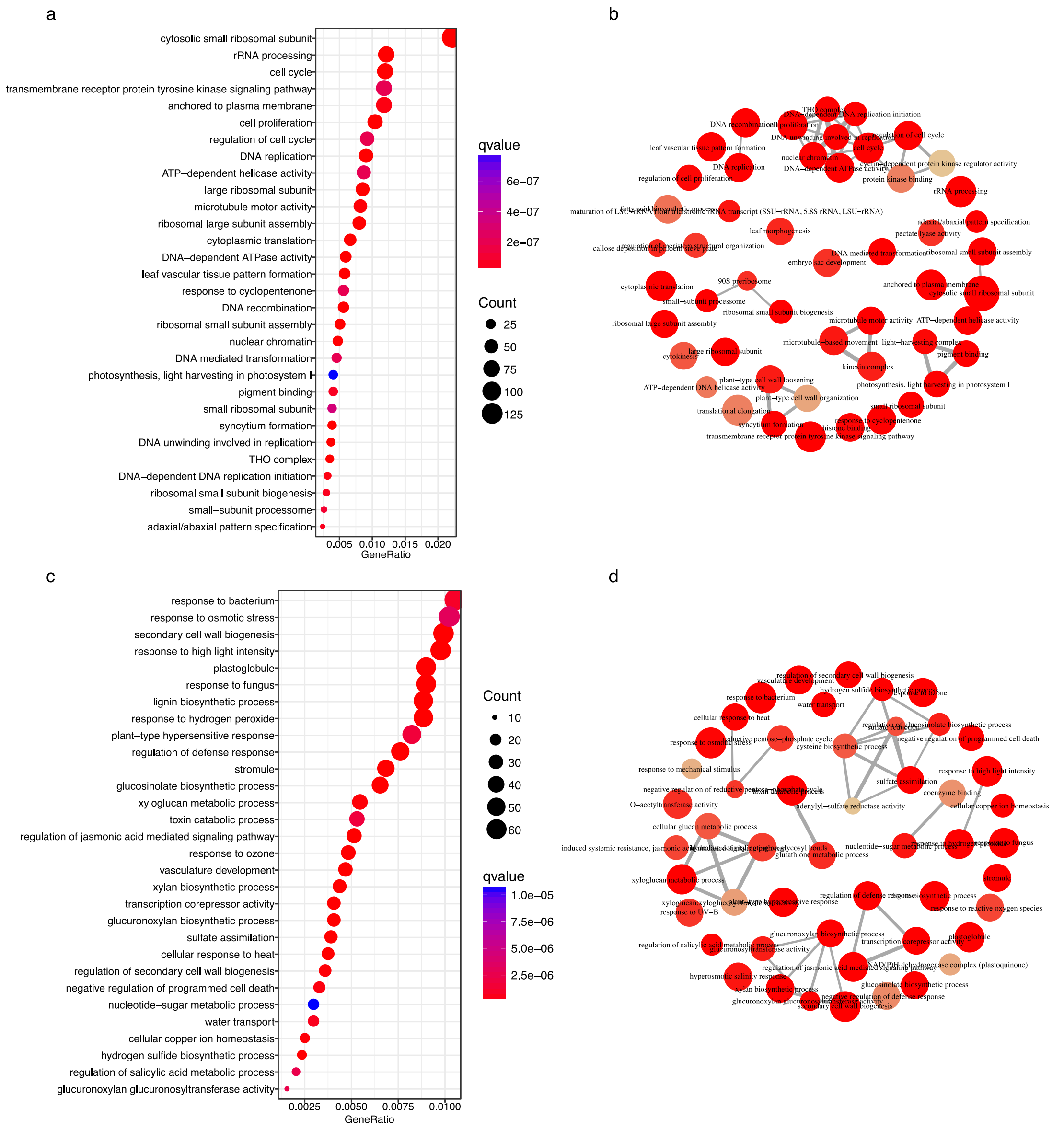

Supplementary Figure 27. GO enrichment analysis results of DEGs between silique pericarps at 7 days after flowering of double-high and double-low *B. napus* accessions. (a) and (c) Overrepresented GO terms in up- and down-regulated DEGs, respectively. (b) and (d) Enrichment results of up- and down-regulated DEGs, respectively. Only the top 30 significantly enriched GO terms were included in this figure.

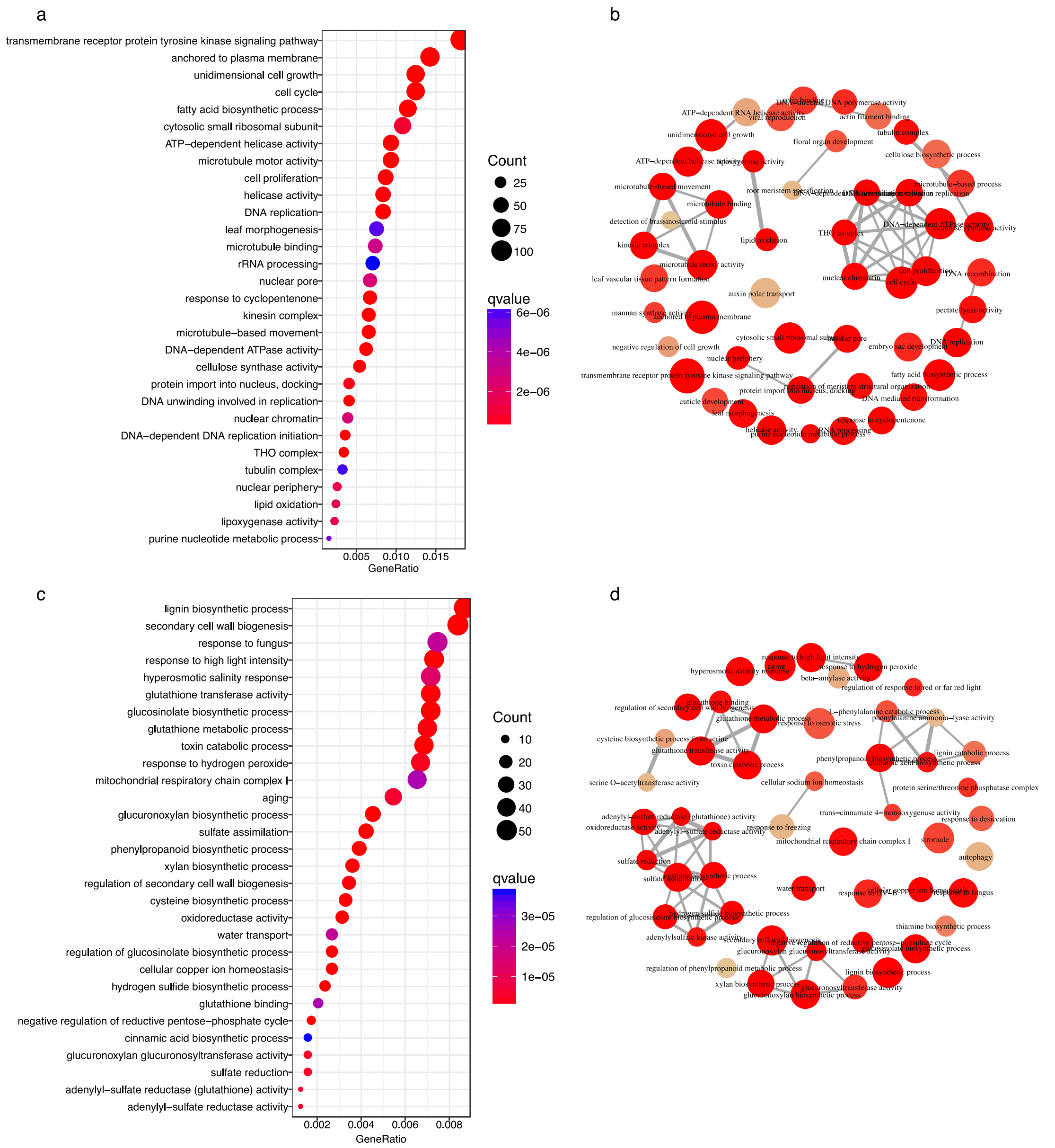

Supplementary Figure 28. GO enrichment analysis results of DEGs between silique pericarps at 10 days after flowering of double-high and double-low *B. napus* accessions. (a) and (c) Overrepresented GO terms in up- and down-regulated DEGs, respectively. (b) and (d) Enrichment results of up- and down-regulated DEGs, respectively. Only the top 30 significantly enriched GO terms were included in this figure.

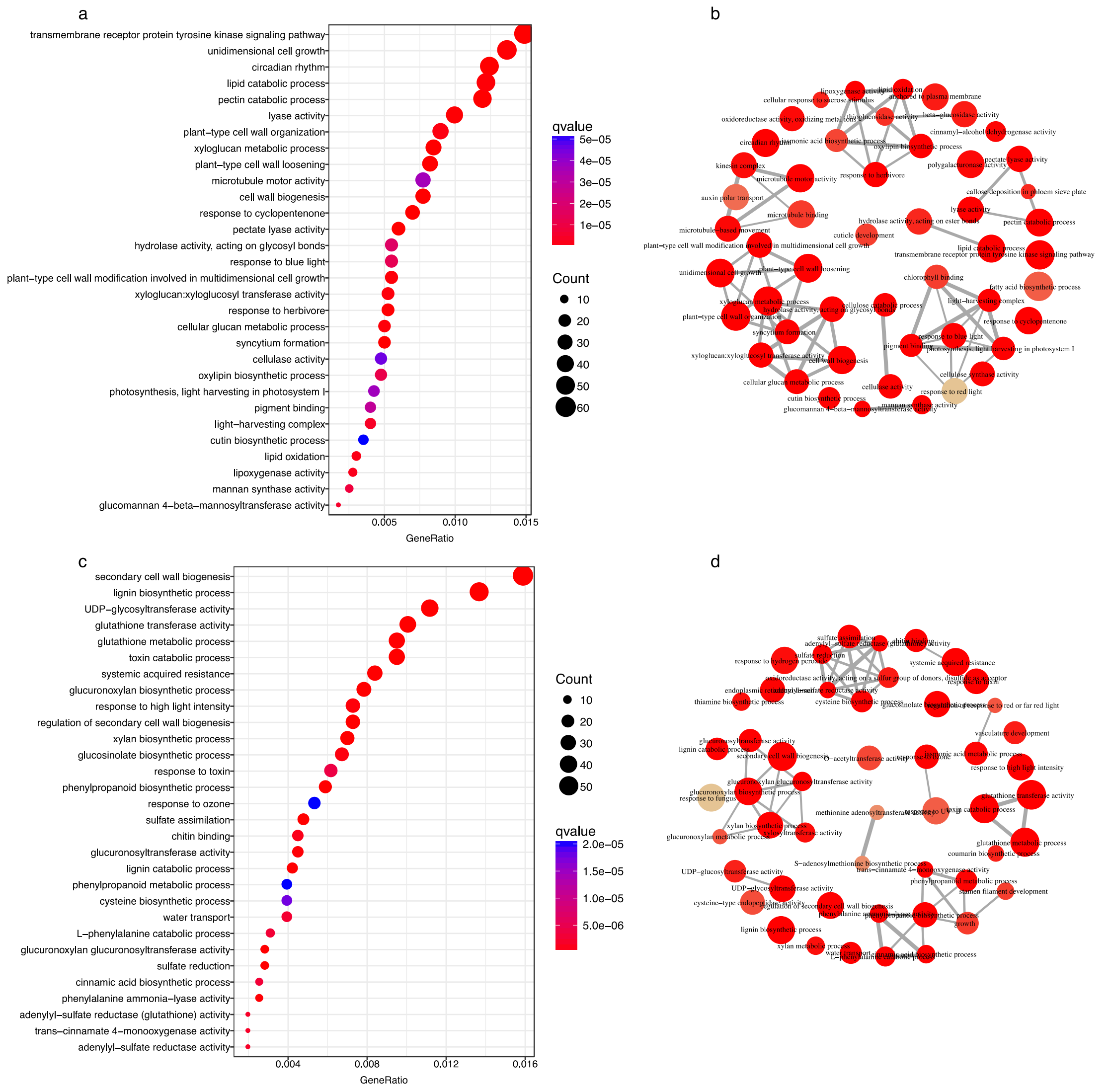

Supplementary Figure 29. GO enrichment analysis results of DEGs between silique pericarps at 14 days after flowering of double-high and double-low *B. napus* accessions. (a) and (c) Overrepresented GO terms in up- and down-regulated DEGs, respectively. (b) and (d) Enrichment results of up- and down-regulated DEGs, respectively. Only the top 30 significantly enriched GO terms were included in this figure.

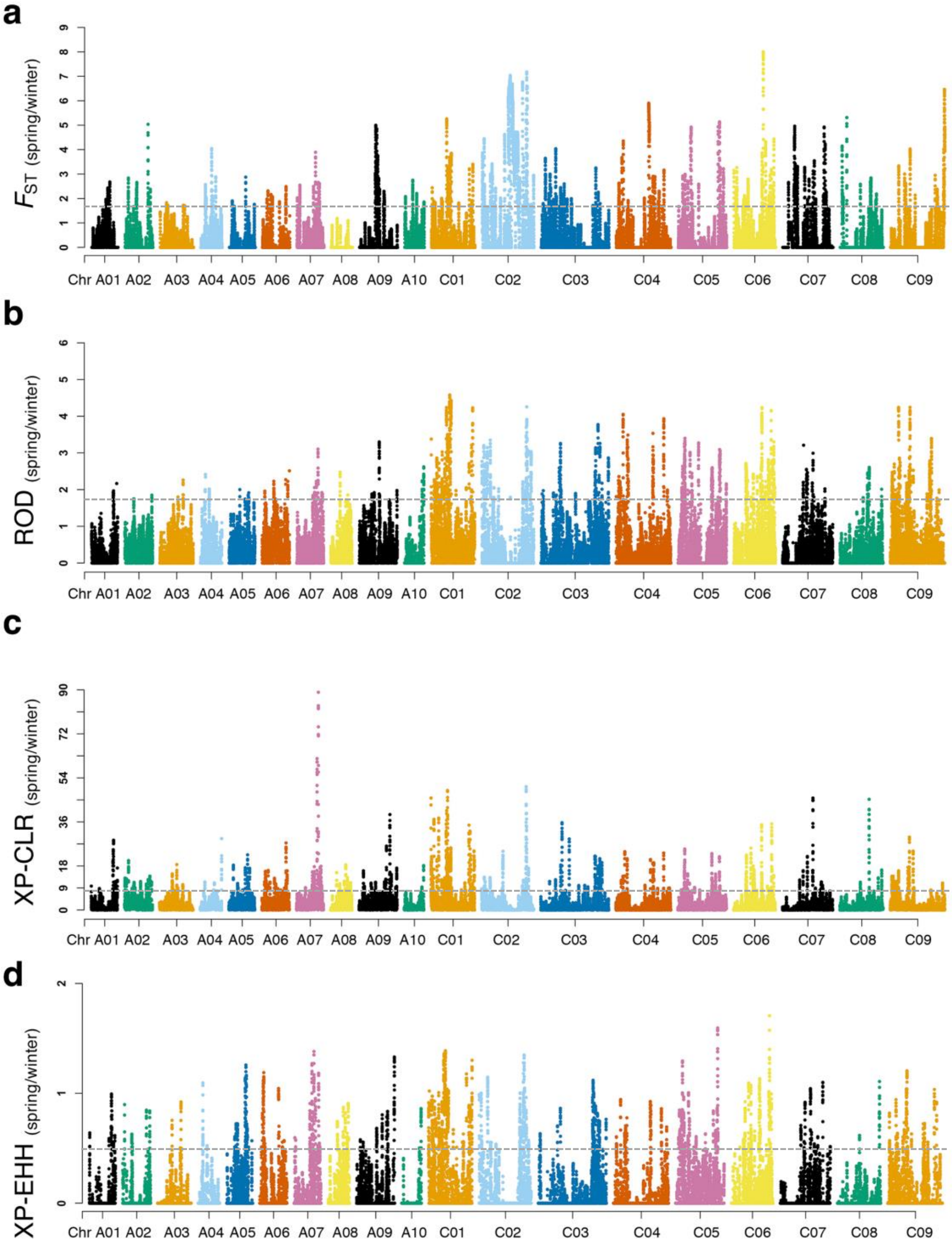

Supplementary Figure 30. Whole-genome scanning of ecotype improvement-selection signatures between spring and winter ecotype of *B. napus*. Ecotype improvement-selection signals detected using (a) F_{ST}, (b) ROD, (c) XPCLR, and (d) XP-EHH comparisons.

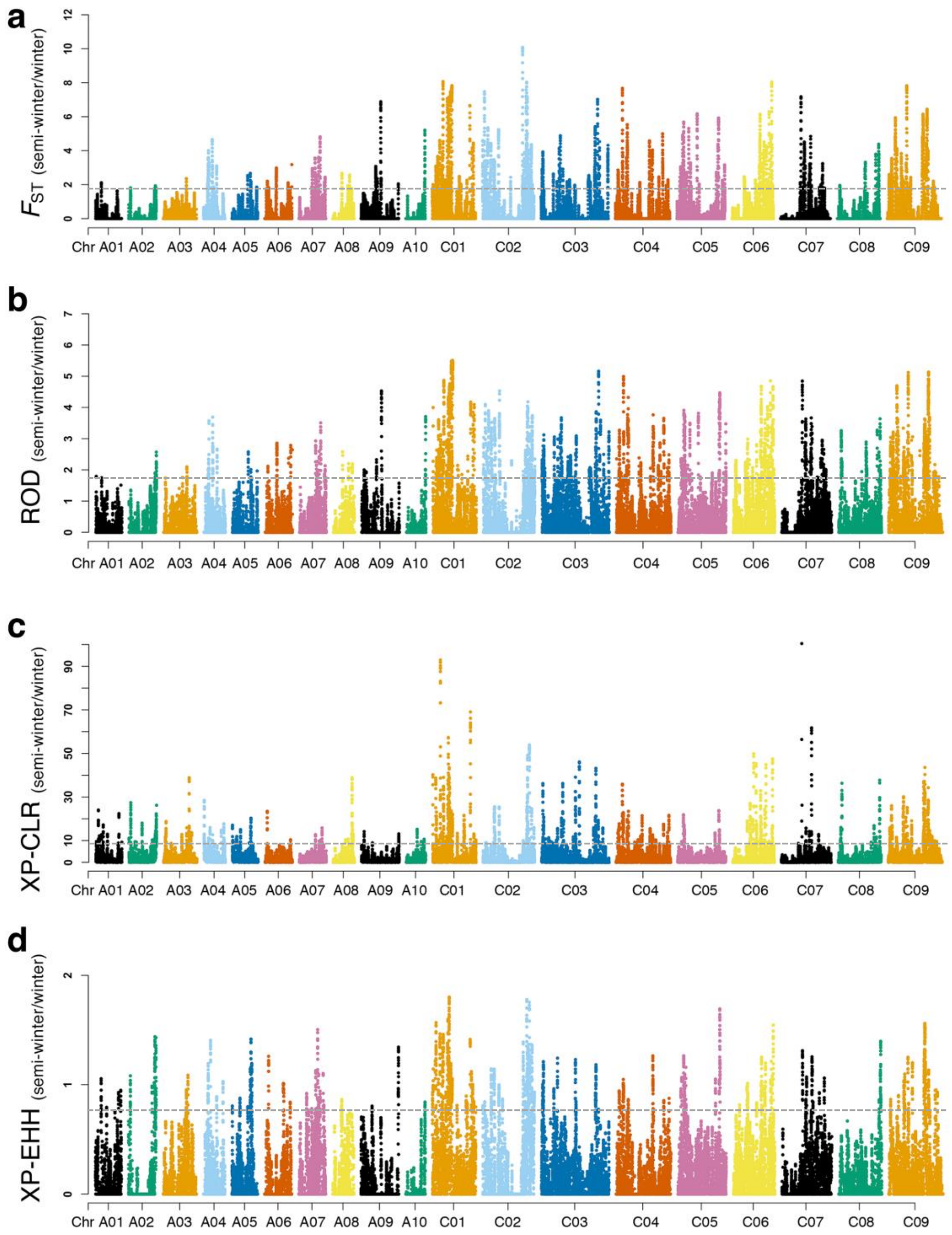

Supplementary Figure 31. Whole-genome scanning of ecotype improvement-selection signatures between semi-winter and winter ecotype of *B. napus*. Ecotype improvement-selection signals detected using (a) F_{ST}, (b) ROD, (c) XPCLR, and (d) XP-EHH comparisons.

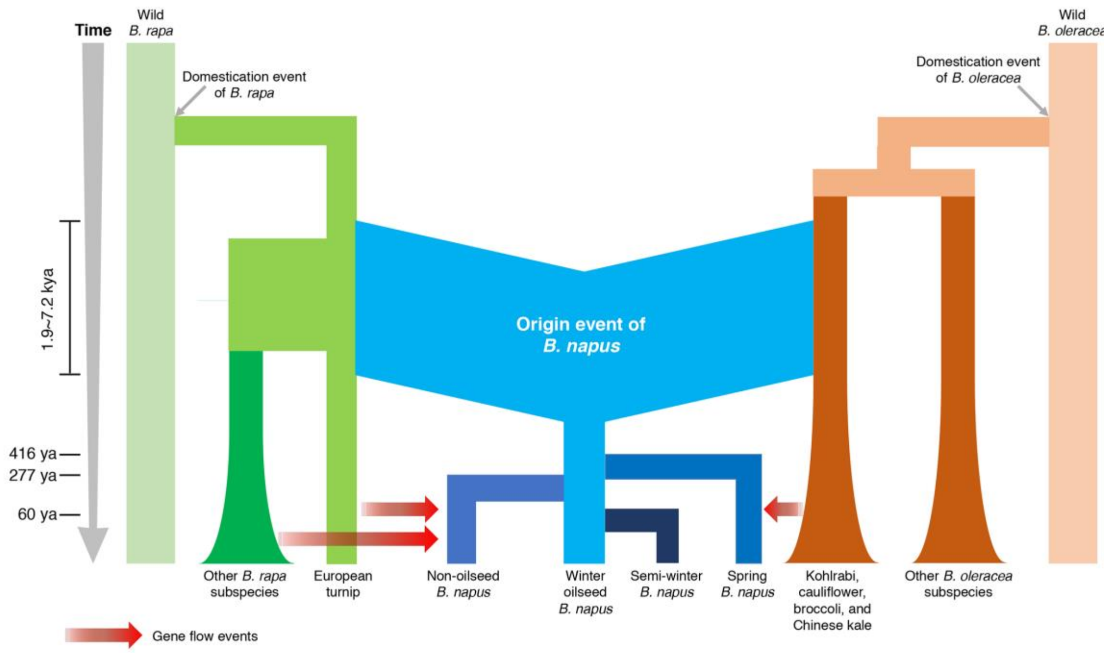

Supplementary Figure 32. Proposed model for origin and evolutionary history of *B. napus*. The origin time of *B. napus*, and divergence times of spring, semi-winter and non-oilseed *B. napus* from winter oilseed *B. napus* were estimated using SMC++[4]. Gene flow events were estimated using fastsimcoal2[3] and marked with red arrows.

Supplementary Table 1. Classification of the 588 *B. napus* accessions.

Improvement	Group	Asia	Europe	North America	Oceania	Total
SSI	Landrace	28	23	3	3	57
	Improved cultivar	55	19	0	1	74
Seed quality	Double high	102	26	1	3	132
	Double low	207	51	7	2	267
Ecotype	Winter	14	53	0	0	67
	Spring	12	46	13	7	78
	Semi-winter	440	3	0	0	443
Usage	Oil	462	92	13	6	573
	Vegetable	4	0	0	0	4
	Fodder	0	10	0	1	11

Supplementary Table 2. Comparison of LD decay among different groups of materials.

Comparison	Classification	BraA (kb)		BolC (kb)		A subgenome of Bna (kb)		C subgenome of Bna (kb)	
		r^2 = 0.1	r^2 = 0.3	r^2 = 0.1	r^2 = 0.3	r^2 = 0.1	r^2 = 0.3	r^2 = 0.1	r^2 = 0.3
Organism	*B. rapa*	25.50	2.10	NC	NC	NC	NC	NC	NC
	B. oleracea	NC	NC	432.20	27.90	NC	NC	NC	NC
	B. napus	179.60	4.30	1,172.40	121.40	322.70	19.30	–	1,365.30
Ecotype	Winter	167.70	6.90	767.40	67.40	362.30	31.90	1,356.10	411.00
	Spring	185.10	6.20	1,307.70	109.00	383.40	34.80	–	1,634.40
	Semi-winter	239.90	4.50	1,198.40	108.70	411.00	31.70	–	3,142.50
Usage	Oilseed	263.80	4.50	1,285.60	109.50	438.90	33.30	–	3,592.50
	Fodder	2,127.00	21.30	–	160.70	4,686.80	98.40	4,659.10	641.40
	Vegetable	–	216.40	–	1,381.00	–	3,093.80	–	3,032.10
SSI	Landrace	NC	NC	NC	NC	393.10	23.80	4,743.50	660.30
	Improved Cultivar	NC	NC	NC	NC	417.30	32.70	–	1,365.30

BraA denotes the SNP set called from *B. napus* and *B. rapa* accessions; BolC is the SNP set called from *B. napus* and *B. oleracea* accessions (Methods). Negative sign indicates that the LD does not drop below the threshold of r^2 = 0.1 or 0.3; NC indicates no calculation, since the LD decay is not accurate calculated from corresponding SNP sets.

Supplementary References

1. Gutenkunst, R. N., Hernandez, R. D., Williamson, S. H. & Bustamante, C. D. Inferring the joint demographic history of multiple populations from multidimensional SNP frequency data. *PLoS Genet.* **5,** e1000695 (2009).
2. Chalhoub, B. *et al.* Early allopolyploid evolution in the post-Neolithic *Brassica napus* oilseed genome. *Science* **345,** 950–953 (2014).
3. Excoffier, L., Dupanloup, I., Huerta-Sánchez, E., Sousa, V. C. & Foll, M. Robust demographic inference from genomic and SNP data. *PLoS Genet.* **9,** e1003905 (2013).
4. Terhorst, J., Kamm, J. A. & Song, Y. S. Robust and scalable inference of population history from hundreds of unphased whole genomes. *Nat. Genet.* **49,** 303–309. (2017).
