# Supplementary material for: Whole-genome resequencing reveals Brassica napus origin and genetic loci involved in its improvement
Source: Nat Commun. 2019 Mar 11;10:1154. doi: 10.1038/s41467-019-09134-9 (PMC6411957; doi:10.1038/s41467-019-09134-9)
Supplement: Supplementary file 3 — Reporting Summary [file 41467_2019_9134_MOESM3_ESM.pdf]

nature research

Corresponding author(s): Kun Lu

Last updated by author(s): Feb 20, 2019

Reporting Summary

Nature Research wishes to improve the reproducibility of the work that we publish. This form provides structure for consistency and transparency in reporting. For further information on Nature Research policies, see Authors & Referees and the Editorial Policy Checklist.

Statistics

For all statistical analyses, confirm that the following items are present in the figure legend, table legend, main text, or Methods section.

n/a	Confirmed	
☐	☒	The exact sample size (*n*) for each experimental group/condition, given as a discrete number and unit of measurement
☐	☒	A statement on whether measurements were taken from distinct samples or whether the same sample was measured repeatedly
☐	☒	The statistical test(s) used AND whether they are one- or two-sided<br>*Only common tests should be described solely by name; describe more complex techniques in the Methods section.*
☒	☐	A description of all covariates tested
☒	☐	A description of any assumptions or corrections, such as tests of normality and adjustment for multiple comparisons
☐	☒	A full description of the statistical parameters including central tendency (e.g. means) or other basic estimates (e.g. regression coefficient) AND variation (e.g. standard deviation) or associated estimates of uncertainty (e.g. confidence intervals)
☐	☒	For null hypothesis testing, the test statistic (e.g. *F*, *t*, *r*) with confidence intervals, effect sizes, degrees of freedom and *P* value noted<br>*Give P values as exact values whenever suitable.*
☒	☐	For Bayesian analysis, information on the choice of priors and Markov chain Monte Carlo settings
☒	☐	For hierarchical and complex designs, identification of the appropriate level for tests and full reporting of outcomes
☒	☐	Estimates of effect sizes (e.g. Cohen's *d*, Pearson's *r*), indicating how they were calculated

Our web collection on statistics for biologists contains articles on many of the points above.

Software and code

Policy information about availability of computer code

Data collection	No software was used for data collection.
Data analysis	No commercial and custom codes were used in our study. Only freely available bioinformatic softwares were used in this study.

For manuscripts utilizing custom algorithms or software that are central to the research but not yet described in published literature, software must be made available to editors/reviewers. We strongly encourage code deposition in a community repository (e.g. GitHub). See the Nature Research guidelines for submitting code & software for further information.

Data

Policy information about availability of data

All manuscripts must include a data availability statement. This statement should provide the following information, where applicable:

- Accession codes, unique identifiers, or web links for publicly available datasets
- A list of figures that have associated raw data
- A description of any restrictions on data availability

The raw sequencing data have been deposited in the NCBI database under BioProject accession codes PRJNA358784 [https://www.ncbi.nlm.nih.gov/bioproject/PRJNA358784] and PRJNA430009 [https://www.ncbi.nlm.nih.gov/bioproject/PRJNA430009], and in the BIG Data Center under BioProject accession codes PRJCA000376 [http://bigd.big.ac.cn/bioproject/browse/PRJCA000376] and PRJCA001246 [http://bigd.big.ac.cn/bioproject/browse/PRJCA001246]. The source data underlying Figs. 1–5, as well as Supplementary Figs. 1–10 and 12–31 are available in the figshare with the identifier [doi: 10.6084/m9.figshare.7737044].

Field-specific reporting

Please select the one below that is the best fit for your research. If you are not sure, read the appropriate sections before making your selection.

☒ Life sciences ☐ Behavioural & social sciences ☐ Ecological, evolutionary & environmental sciences

For a reference copy of the document with all sections, see nature.com/documents/nr-reporting-summary-flat.pdf

Life sciences study design

All studies must disclose on these points even when the disclosure is negative.

Sample size	For whole genome re-sequencing, a total of 608 B. napus accessions (588 different accessions, including 20 biological replicates) were used. For RNA-Seq data of 11 tissues in two different B. napus accessions, two biological replicates were used.
Data exclusions	In our analysis, the low quality sequencing reads were removed before our genomic variation detection. SNPs with a MAF of < 5% were also filtered. These exclusions were important for results accuracy.
Replication	We used different methods to evaluate our evolutionary models and accuracy of SNP data.
Randomization	To measure the 11 important traits, all B. napus accessions were cultivated in field trials in Beibei, Chongqing, China (29°45′ N, 106°22′ E, 238.57 m) during the 2013 and 2014 growing seasons. A randomized complete block design with three replications was employed. Each accession was grown in three rows of 10 plants per row, with a distance of 20 cm between plants and 30 cm between rows.
Blinding	Blinding was not necessary to our study.

Reporting for specific materials, systems and methods

We require information from authors about some types of materials, experimental systems and methods used in many studies. Here, indicate whether each material, system or method listed is relevant to your study. If you are not sure if a list item applies to your research, read the appropriate section before selecting a response.

Materials & experimental systems

n/a	Involved in the study	
☒	☐	Antibodies
☒	☐	Eukaryotic cell lines
☒	☐	Palaeontology
☒	☐	Animals and other organisms
☒	☐	Human research participants
☒	☐	Clinical data

Methods

n/a	Involved in the study	
☒	☐	ChIP-seq
☒	☐	Flow cytometry
☒	☐	MRI-based neuroimaging
