# Supplementary material for: Whole-genome resequencing reveals Brassica napus origin and genetic loci involved in its improvement
Source: Nat Commun. 2019 Mar 11;10:1154. doi: 10.1038/s41467-019-09134-9 (PMC6411957; doi:10.1038/s41467-019-09134-9)
Supplement: Supplementary file 4 — Description of Additional Supplementary Files [file 41467_2019_9134_MOESM4_ESM.docx]

**Description of Additional Supplementary Files**

File Name: Supplementary Data 1

Description: Detailed information of the 588 *B. napus* accessions (608 samples).

File Name: Supplementary Data 2

Description: Statistics of SNPs identified in this study.

File Name: Supplementary Data 3

Description: Statistics of indels identified in this study.

File Name: Supplementary Data 4

Description: Validation of SNP calling accuracy by Sanger sequencing.

File Name: Supplementary Data 5

Description: Determination of the optimal model used for ML tree construction of *B. napus*.

File Name: Supplementary Data 6

Description: Determination of the optimal model used for ML tree construction of *B. napus* A subgenome.

File Name: Supplementary Data 7

Description: Determination of the optimal model used for ML tree construction of *B. napus* C subgenome.

File Name: Supplementary Data 8

Description: Average nucleotide-diversity in sliding windows of AA and AL.

File Name: Supplementary Data 9

Description: Average nucleotide-diversity in sliding windows of CA and CL.

File Name: Supplementary Data 10

Description: Average *F*_ST_ in sliding windows between AA and AL.

File Name: Supplementary Data 11

Description: Average *F*_ST_ in sliding windows between CA and CL.

File Name: Supplementary Data 12

Description: Average *F*_ST_ in sliding windows between landraces and improved *B. napus* cultivars.

File Name: Supplementary Data 13

Description: Common FSI-selection windows overlapped with high ROD and *F*_ST_ between AA and AL.

File Name: Supplementary Data 14

Description: Common FSI-selection windows overlapped with high ROD and *F*_ST_ between CA and CL.

File Name: Supplementary Data 15

Description: Candidate genes in the high ROD _(AL/AA)_ and *F*_ST (AL/AA)_ overlapped outlier regions.

File Name: Supplementary Data 16

Description: Candidate genes in the high ROD _(CL/CA)_ and *F*_ST (CL/CA)_ overlapped outlier regions.

File Name: Supplementary Data 17

Description: GO categories of BP enriched in both high ROD _(AL/AA)_ and *F*_ST_ _(AL/AA)_ overlapped outlier regions.

File Name: Supplementary Data 18

Description: GO categories of BP enriched in both high ROD _(CL/CA)_ and *F*_ST_ _(CL/CA)_ overlapped outlier regions.

File Name: Supplementary Data 19

Description: Overlapped improvement-selection windows detected by at least three methods between landraces and improved *B. napus* cultivars.

File Name: Supplementary Data 20

Description: Candidate genes in the overlapped improvement-selection outliers detected by at least three methods between landraces and improved *B. napus* cultivars.

File Name: Supplementary Data 21

Description: GO categories of BP enriched in the overlapped improvement-selection outliers detected by at least three methods between landraces and improved *B. napus* cultivars.

File Name: Supplementary Data 22

Description: Overlapped improvement-selection windows detected by at least three methods between double-high and double-low *B. napus* cultivars.

File Name: Supplementary Data 23

Description: Candidate genes in the overlapped improvement-selection outliers detected by at least three methods between double-high and double-low *B. napus* cultivars.

File Name: Supplementary Data 24

Description: GO categories of BP enriched in the overlapped improvement-selection outliers detected by at least three methods between double-high and double-low *B. napus* cultivars.

File Name: Supplementary Data 25

Description: Validation of imputation accuracy by Sanger sequencing.

File Name: Supplementary Data 26

Description: Summary of significantly associated SNP and LD blocks in the GWAS results.

File Name: Supplementary Data 27

Description: Read mapping summary for each sample in *B. napus*.

File Name: Supplementary Data 28

Description: Significantly over-enriched GO terms of DEGs in 11 *B. napus* tissues.

File Name: Supplementary Data 29

Description: Candidate genes improving environmental adaptation and morphogenesis during the FSI of *B. napus*.

File Name: Supplementary Data 30

Description: Overlapped improvement-selection windows detected by at least three methods between *B. napus* winter and semi-winter ecotype.

File Name: Supplementary Data 31

Description: Candidate genes in the overlapped improvement-selection outliers detected by at least three methods between *B. napus* winter and semi-winter ecotype.

File Name: Supplementary Data 32

Description: Overlapped improvement-selection windows detected by at least three methods between *B. napus* winter and spring ecotype.

File Name: Supplementary Data 33

Description: Candidate genes in the overlapped improvement-selection outliers detected by at least three methods between *B. napus* winter and spring ecotype

File Name: Supplementary Data 34

Description: GO categories of BP enriched in the overlapped improvement-selection outliers detected by at least three methods between *B. napus* winter and semi-winter ecotype.

File Name: Supplementary Data 35

Description: GO categories of BP enriched in the overlapped improvement-selection outliers detected by at least three methods between *B. napus* winter and spring ecotype.

File Name: Supplementary Data 36

Description: Candidate flowering-time genes in the overlapped ecotype improvement-selection outliers.

File Name: Supplementary Data 37

Description: Candidate genes associated with seed oil content in the nine FSI-selection signals that overlap with oil content QTLs.
